# Fatty acid conjugation enhances potency of antisense oligonucleotides in muscle

**DOI:** 10.1093/nar/gkz354

**Published:** 2019-05-25

**Authors:** Thazha P Prakash, Adam E Mullick, Richard G Lee, Jinghua Yu, Steve T Yeh, Audrey Low, Alfred E Chappell, Michael E Østergaard, Sue Murray, Hans J Gaus, Eric E Swayze, Punit P Seth

**Affiliations:** 1 Ionis Pharmaceuticals, Medicinal Chemistry, USA; 2 Antisense Drug Discovery, 2855 Gazelle Ct., Carlsbad, CA 92010, USA

## Abstract

Enhancing the functional uptake of antisense oligonucleotide (ASO) in the muscle will be beneficial for developing ASO therapeutics targeting genes expressed in the muscle. We hypothesized that improving albumin binding will facilitate traversal of ASO from the blood compartment to the interstitium of the muscle tissues to enhance ASO functional uptake. We synthesized structurally diverse saturated and unsaturated fatty acid conjugated ASOs with a range of hydrophobicity. The binding affinity of ASO fatty acid conjugates to plasma proteins improved with fatty acid chain length and highest binding affinity was observed with ASO conjugates containing fatty acid chain length from 16 to 22 carbons. The degree of unsaturation or conformation of double bond appears to have no influence on protein binding or activity of ASO fatty acid conjugates. Activity of fatty acid ASO conjugates correlated with the affinity to albumin and the tightest albumin binder exhibited the highest activity improvement in muscle. Palmitic acid conjugation increases ASO plasma C_max_ and improved delivery of ASO to interstitial space of mouse muscle. Conjugation of palmitic acid improved potency of DMPK, Cav3, CD36 and Malat-1 ASOs (3- to 7-fold) in mouse muscle. Our approach provides a foundation for developing more effective therapeutic ASOs for muscle disorders.

## INTRODUCTION

Nucleic acid-based therapeutics represent a distinct drug-discovery platform with the ability to target genes linked to disease that are considered undruggable by classic small molecule approaches (1–3). Several nucleic-acid based drugs are currently approved for clinical use or in late stage of clinical development (3). Conjugation of tri-antennary *N*-acetyl galactosamine enhanced ASO uptake into hepatocytes through ASGR-mediated internalization, improving hepatocyte potency 10- to 60-fold (4). Recent studies showed that conjugation of ASOs to a ligand of the glucagon like peptide-1 receptor (GLP1R) improved ASO uptake into pancreatic ß-cells and enhanced potency >50-fold (5). This was remarkable since pancreatic ß-cells are known to be refractory to ASO uptake (6) and this opens therapeutic opportunities to treat diseases affecting the pancreas such as diabetes. To further expand the utility of antisense technology it is important to improve ASO potency in additional tissues beyond the liver and ß-cells. Therefore, delivery approaches which enhance ASO potency in extrahepatic tissues will be beneficial.

ASOs show robust gene silencing in extrahepatic tissues such as muscle after systemic administration but higher doses are required (6). Development of Ionis DMPK-2.5_Rx_ was recently discontinued because of inadequate therapeutic benefit in short-term clinical trials in patients with myotonic dystrophy type 1 (DM1) (7). We envisage that more potent ASOs with improved muscle delivery technologies would ameliorate poor efficacy of this drug in patients.

Previous work showed that cholesterol conjugated ASOs show increased activity and cellular association through LDL receptor mediated endocytosis (8,9). Wolfrum *et al.* conjugated cholesterol and variety of lipids to siRNA and demonstrated that long-chain fatty acids and cholesterol can facilitate siRNA uptake into cells for effective gene silencing in mice (10). This group also showed that efficient and selective uptake of siRNA conjugates depends on interaction with lipoprotein particles, lipoprotein receptors and other cell-surface receptors (10). There are additional examples where lipid conjugation improved potency of siRNA and ASOs in cells (11) and in mice (12–15). Cholesterol conjugated siRNA also inhibited myostatin mRNA expression in skeletal muscle after systemic administration (16).

Skeletal and cardiac muscle cells rely heavily on the oxidation of long-chain fatty acids for contractile work (17). Fatty acids are transported to muscle tissue *via* the blood either complexed to albumin or covalently bound in triacylglycerols forming the neutral lipid core of circulating triglyceride-rich lipoproteins such as chylomicrons or very low-density lipoproteins (17). The capillary endothelium represents one of the first barriers fatty acids have to traverse on their way from the vascular compartment to skeletal and cardiac muscle cells (17). The mechanism responsible for transmembrane movement of fatty acids is incompletely understood, however recent studies have revealed that interaction of the albumin-fatty acid complex with the endothelial membrane may facilitate fatty acid uptake (17).

Serum albumin is a transport protein for endogenous fatty acids. Albumin is the most abundant plasma protein in human blood (35–50 g/l, Molecular weight 66.5 kDa) (18) and it is synthesized in the liver and released into the vascular space (19). Albumin interacts with multiple cellular receptors such as glycoprotein Gp60, Gp30 and Gp18a, the Megalin/Cubilin complex, and the neonatal Fc receptor (FcRn) (20). The interaction with these receptors are responsible for albumin's recycling, transcytosis and extended half-life (∼19 days) in circulation (20). Albumin contains multiple hydrophobic pockets that bind fatty acids and steroids as well as different drugs (20). Palmitic acid conjugated glucagon-like type-1 (GLP-1) agonist liraglutide (21) and Myristic acid conjugated detemir (22) also utilize the fatty-acid binding properties of albumin to improve their pharmacology.

ASOs with PS linkages are known to bind serum albumin with dissociation constants in the low micromolar range (23). We hypothesized that fatty acid conjugation will improve the binding of PS ASO with serum albumin and facilitate ASO transport across the continuous capillary endothelial in the skeletal and cardiac muscle (24). In this report, we describe results from our detailed investigation of using fatty acid conjugation to enhance ASO activity in muscle. We show that conjugation of palmitic acid enhances affinity of PS ASOs for serum albumin and that these conjugates show enhanced potency in muscle tissues. We also report the detailed SAR of fatty acid-ASO conjugates to identify the optimal fatty acid for enhancing ASO potency.

## MATERIALS AND METHODS

### General method for the synthesis of fatty acid pentafluorophenyl esters 4, 22–28, 31, 56–67

**Scheme 1.**
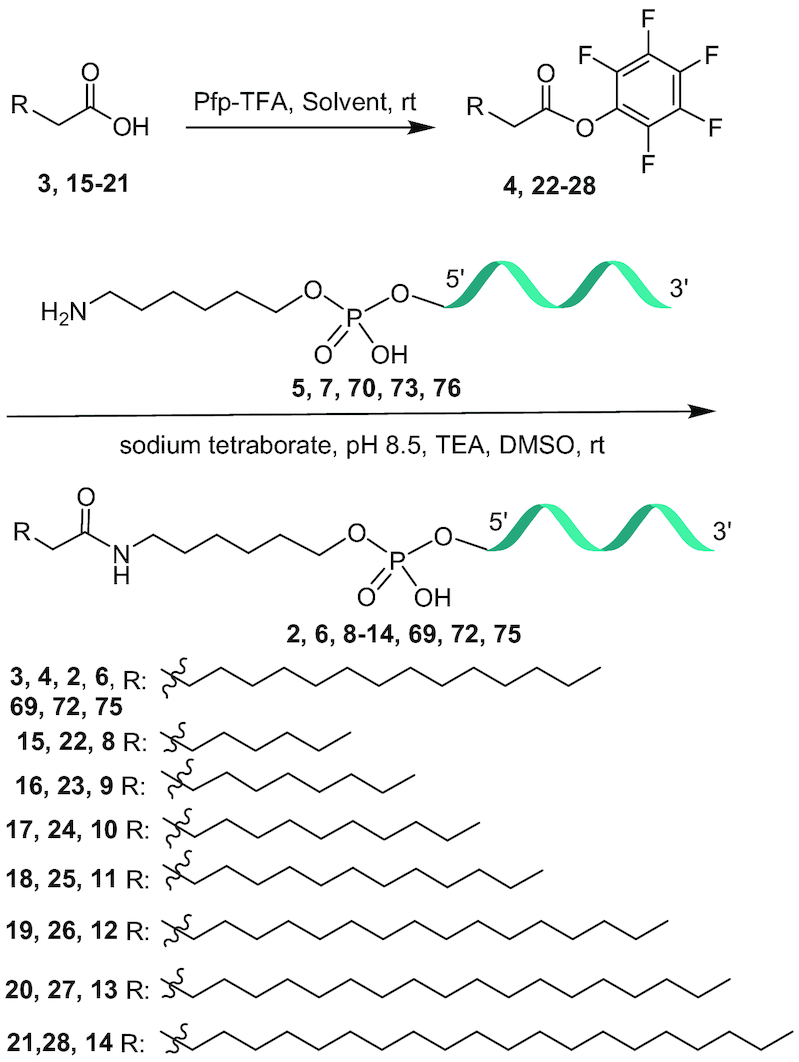
Synthesis of 5′-fatty acid conjugated ASO **2, 6, 8–14, 69, 72** and **75;** ASO **5:** 5′-T^m^CAG_k_^m^C_k_A_k_ T_d_T_d_^m^C_d_ T_d_A_d_A_d_T_d_A_d_ G_d_^m^C_d_ A_k_G_k_^m^C_k_ 3′, ASO **7**: G_k_^m^C_k_ A_k_T_d_T_d_^m^C_d_ T_d_A_d_A_d_T_d_A_d_ G_d_^m^C_d_ A_k_G_k_^m^C_k_, ASO **70**: 5′- T_d_^m^C_d_A_d_A_k_G_k_G_k_A_d_T_d_ A_d_T_d_ G_d_G_d_A_d_A_d_^m^C_d_^m^C_d_ A_k_A_k_A_k-_3′, ASO **73**: 5′-A_k_^m^C_k_A_k_ A_d_T_d_ A_d_A_d_ A_d_T_d_A_d_^m^C_d_^m^C_d_G_d_ A_k_G_k_G_k_-3′, ASO **76**: 5′-^m^C_k_^m^C_k_^m^C_k_ T_d_ T_d_T_d_A_d_ T_d_T_d_ G_d_^m^C_d_A_d_G_d_^m^C_k_A_k_^m^C_k_, k: cEt BNA, d: DNA, ^m^C: 5-methyl cytidine, Backbone all PS, underline PO.

**Scheme 2.**
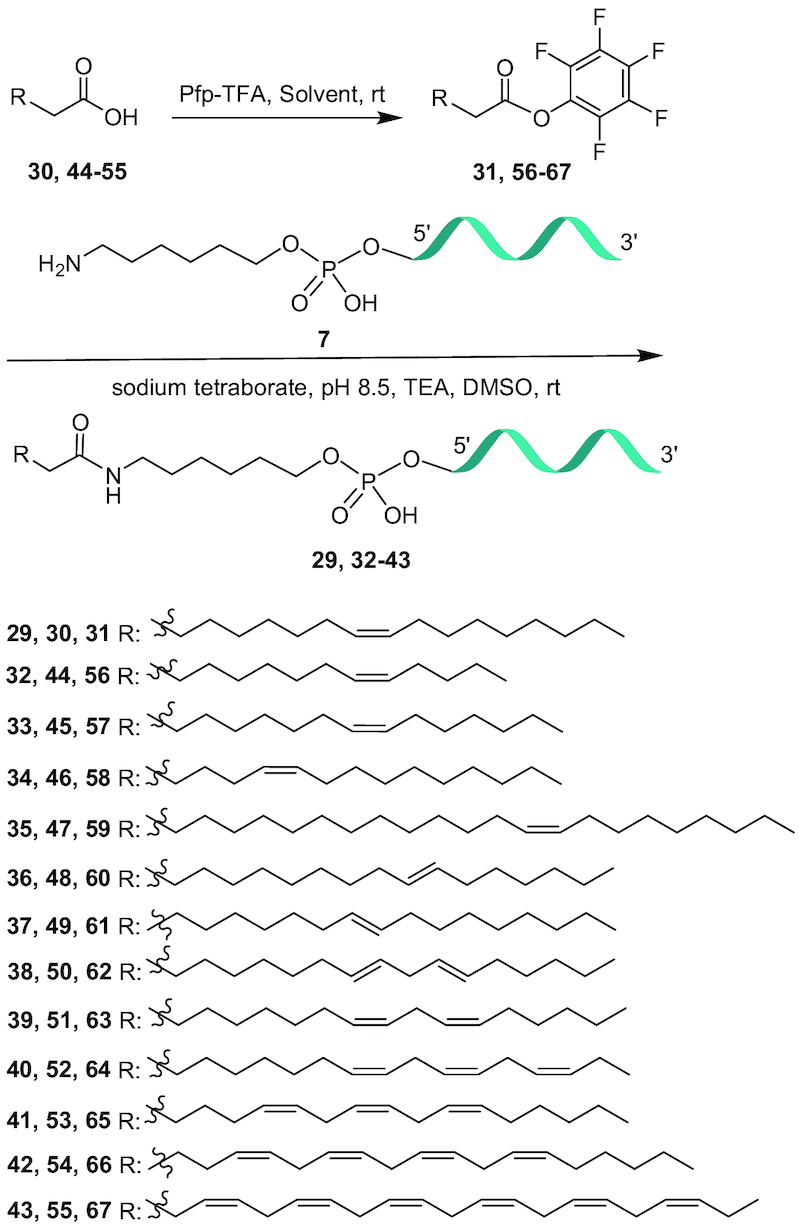
Synthesisof 5’-unsaturated fatty acid conjugated ASO **29, 32-43**.

Fatty acids **3, 15**–**21, 30, 44**–**55** (1 mmol, Schemes 1 and 2), TEA (2.25 ml, 16 mmol) were dissolved in DCM (1 ml/mmol) and pentafluorophenyl trifluoroacetate (4 mmol) was added. Stirred the reaction mixture at room temperature for 1 h. The reaction mixture was diluted with dichloromethane (6 ml/mmol) and washed with aqueous saturated NaHCO_3_ (5 ml/mmol) solution and 1 N NaHSO_4_ (5 ml/mmol) solution. The organic layer was separated, dried (Na_2_SO_4_), filtered and concentrated under reduced pressure. The crude product was purified by silica gel column chromatography and eluted with solvents (See Supporting Information) to yield **4, 22–28, 31, 56–67** in 70–82% isolated yield. All the compounds were characterized by ^1^H and ^13^C NMR spectroscopic analysis (Supporting Information).

### General method for the synthesis of fatty acid ASO conjugates 2, 6, 8–14, 29, 32–43, 69, 72, 75

To a solution of 5′-hexylamino ASOs **5, 7, 70, 73, 76** (Schemes 1 and 2) in 0.1 M sodium tetraborate buffer, pH 8.5 (2 mM) a solution of fatty acid PFP ester **4, 22–28, 31, 56–67** (3–9 mole equivalent, Scheme 1 and 2) dissolved in DMSO (40 mM), acetonitrile (20 mM) and triethylamine was added and the reaction mixture was stirred at room temperature for 3–18 h. The reaction mixture was diluted with water and purified by HPLC on a strong anion exchange column (GE Healthcare Bioscience, Source 30Q, 30 μm, 2.54 × 8 cm, A = 100 mM ammonium acetate in 30% aqueous CH_3_CN, B = 1.5 M NaBr in A, 0–60% of B in 60 min, flow 14 ml min^−1^). HPLC fractions containing full length ASO conjugates (analyzed by LC MS) were pooled together and diluted three fold volume with water and desalted by HPLC on a reverse phase column to yield the 5′-fatty acid conjugated ASOs **2, 6, 8–14, 29, 32–43, 69, 72, 75** in an isolated yield of 50–78%. The ASOs were characterized by ion-pair-HPLC–MS analysis with Agilent 1100 MSD system (Supporting Information).

### *N*-(6-hydroxyhexyl)palmitamide 78

To a solution of palmitic acid **3** (10.0 g, 39.0 mmol) and triethylamine (16.3 ml, 117.0 mmol) in dichloromethane (800 ml) pentafluorophenyl trifluoroacetate (10.1 ml, 58.5 mmol) was added dropwise at room temperature. To this 6-aminohexanol **79** (5.48 g, 46.8 mmol) and dichloromethane (200 ml) were added. The reaction mixture was stirred at room temperature for 12 h. Compound **78** (11.6 g, 84%) precipitated from the solution and collected by filtration. ^1^H NMR (300MHz, CDCl_3_) δ: 5.42 (br s, 1H), 3.65 (t, *J* = 6.3 Hz, 2H), 3.29-3.23 (m, 2H), 2.16 (t, *J* = 6.3 Hz, 2H), 1.70–1.46 (m, 6H), 1.45–1.19 (m, 28H), 0.89 (t, *J* = 6.3 Hz, 3H); ^13^C NMR (75MHz, CDCl_3_) δ: 173.11, 62.71, 39.24, 36.95, 32.56, 31.91, 29.70, 29.68, 29.64, 29.61, 29.49, 29.35, 29.32, 26.49, 25.82, 25.27, 22.68, 14.09. LRMS (ESI) *m/z* calcd for C_22_H_46_NO_2_ [M + H]^+^ 356.6, found 356.3.

### 2-Cyanoethyl (6-palmitamidohexyl) diisopropylphosphoramidite 77

To compound **78** (7.3 g, 20.5 mmol) DMF (50 ml) and THF (40 ml) were added. The mixture was heated at 50°C to get a clear solution. To this solution, tetrazole (1.15 g, 16.40 mmol) and 1-methylimidazole (0.41 ml, 5.13 mmol) were added and the reaction mixture was cooled in an ice bath (Note: solution became cloudy). 2-Cyanoethyl-*N*,*N*,*N*',*N*'-tetraisopropylphosphorodiamidite (9.78 ml, 30.8 mmol) was added and the reaction mixture was removed from the ice bath and stirred at room temperature for 12 h. The reaction mixture was then diluted with ethyl acetate (50 ml), washed with saturated NaHCO_3_ solution (50 ml) followed by brine (50 ml), and then dried (Na_2_SO_4_), filtered and concentrated under reduce pressure. The residue obtained was purified by silica gel column chromatography and eluted with 30% ethyl acetate in hexanes containing 1% triethylamine to yield **77** (10.7 g, 93%). ^31^P NMR (121MHz, CDCl_3_) δ: 147.49; HRMS (ESI) *m/z* calcd for C_33_H_66_N_3_O_3_P [M + H]^+^ 584.4915, found 584.4943.

### Fluorescence polarization assay

Fluorescence polarization experiments were performed using ALEXA647-labeled ASOs. Measurements were performed in 1× phosphate-buffered saline (PBS). The assay was setup in 96-well Costar plates (black flat-bottomed non-binding) purchased from Corning, NY, USA. Binding was evaluated by adding ALEXA 647-labeled ASOs to yield 2 nM concentration to each well containing 100 μl of protein from sub nM to low mM concentration. Readings were taken using the Tecan (Baldwin Park, CA, USA) InfiniteM1000 Pro instrument (λ_ex_ = 635 nm, λ_em_ = 675 nm). Using polarized excitation and emission filters, the instrument measures fluorescence perpendicular to the excitation plane (the ‘P-channel’) and fluorescence that is parallel to the excitation plane (the ‘S-channel’), and then it calculates FP in millipolarization units (mP) as follows: mP = [(S – P * G)/(S + P × G)] × 1000. The ‘G-factor’ is measured by the instrument as a correction for any bias toward the P channel. Polarization values of each ALEXA 647-labeled ASO in 1× PBS at 2 nM concentration were subtracted from each measurement. *K*_d_ values were calculated with GraphPad Prism 5 software (GraphPad Software, La Jolla, CA, USA) using non-linear regression for curve fit assuming one binding site.

### Animal treatment

Animal experiments were conducted in accordance with the American Association for the Accreditation of Laboratory Animal Care guidelines and were approved by the Animal Welfare Committee (Cold Spring Harbor Laboratory's Institutional Animal Care and Use Committee guidelines). The animals were housed in micro-isolator cages on a constant 12 h light–dark cycle with controlled temperature and humidity and were given access to food and water ad libitum. Blood was collected by cardiac puncture exsanguination with K_2_-EDTA (Becton Dickinson Franklin Lakes, NJ, USA) and plasma separated by centrifugation at 10 000 rcf for 4 min at 4°C. Plasma transaminases were measured using a Beckman Coulter AU480 analyzer. Tissues were collected, weighed, flash frozen on liquid nitrogen and stored at −60°C. Reduction of target mRNA expression was determined by real time RT-PCR using StepOne RT–PCR machines (Applied Biosystems). Briefly, RNA was extracted from ∼50 to 100 mg tissue from each mouse using PureLink Pro 96 Total RNA Purification Kit (LifeTechnologies, Carlsbad, CA, USA) and mRNA was measured by qRT-PCR using Express One-Step SuperMix qRT-PCR Kit (Life Technologies, Carlsbad, CA, USA). Primers and probes for the PCR reactions were obtained from Integrated DNA technologies (IDT). The assay is based on a target-specific probe labeled with a fluorescent reporter and quencher dyes at opposite ends. The probe is hydrolyzed through the 5′-exonuclease activity of Taq DNA polymerase, leading to an increasing fluorescence emission of the reporter dye that can be detected during the reaction. Target RNA levels were normalized to cyclophilin mRNA expression or total mRNA as measured by Ribogreen.

### Malat-1 mouse protocol

Malat-1 ASOs **1**–**2, 6, 8–14, 29** and **32–43** subcutaneously administrated to 8-week-old male C57BL/6 mice (Jackson Laboratories) at various doses. RNA was extracted using Invitrogen PureLink Pro 96 Total RNA purification kit after 5 days of last dose, mice were sacrificed for mouse heart, quadriceps, liver and kidney Malat-1 RNA quantification. PCR was done using Invitrogen Express One-Step qRT-PCR kit using primer probe set MALAT1 5′-TGGGTTAGAGAAGGCGTGTACTG-3′ for the forward primer, 5′-TCAGCGGCAACTGGG AAA-3′ for the reverse primer, and 5′- CGTTGGCACGACACC TTCAGGGACT-3′ for the probe. Target RNA levels were normalized to cyclophilin mRNA expression. The sequences for the primers and probe used for mouse Cyclophilin A are 5′-TCGCCGCTTGCTGCA-3′ for the forward primer, 5′-ATCGGCCGTGATGTCGA-3′ and 5′- CCATGGTCAACCCCACCG TGTTCX-3′ for the probe with 5′ fluorescein and 3′ TAMRA.

### CD36 mouse protocol

CD36 ASOs **68**–**69** intravenous administrated to six to 8-week-old female C57BL/6 mice (Jackson Laboratories) at 1, 3 and 9 μmol/kg once a week for 3 weeks. Mice liver, kidney, heart and quadriceps were harvested 5 days after the last dose for CD36 mRNA quantitation using Invitrogen Express One-Step qRT-PCR kit. The sequences for the primers and probe used for mouse CD36 are 5′-TCCAGCCAATGCCTTTGC-3′ for the forward primer, 5′-GAGATTACTTTTTCAGTGCAGAA-3′ for the reverse primer, and 5′-TCACCCCTCCAGAA TCCAGACAACCAT-3′ for the probe with 5′ fluorescein and 3′ TAMRA. Target RNA levels were normalized to total RNA (Ribogreen© assessment).

### DMPK mouse protocol

ASOs administrated subcutaneously to 6-week-old male Balb/c mice at ASO **71** at 1.84, 3.68 and 7.36 μmol/kg and ASO **72** at 0.85, 1.70 and 3.40 μmol/kg once a week for 3.5 weeks. Heart and quadriceps were harvested after 2 days after the last dose. DMPK mRNA was quantitated using Invitrogen Express One-Step qRT-PCR kit using DMPK forward primer: 5′-GACATATGCCAAGATTGTGCACTAC-3′, reverse primer: 5′-CACGAAT GAGG TCCTG AGCTT-3′ and 5′ fluorescein and 3′ TAMRA probe 5′-AACACTTGT CG CTG CCGCTGGC. Target RNA levels were normalized to total RNA (Ribogreen© assessment).

### Caveolin-3 (Cav3) mouse protocol

ASOs **74**–**75** administrated subcutaneously to 6- to 8-week old male C57BL/6 mice at 1.68, 5 and 15 μmol/kg once a week for two weeks. Heart and quadriceps were harvested after 4 days after the last dose. Cav3 mRNA was quantitated using Invitrogen Express One-Step qRT-PCR kit using Cav3 forward primer: 5′-CATCAAGGACATTCACTGCAAG-3′, reverse primer: 5′-CTCCGCAATCACGTCTTCA-3′ and probe 5′- AACCGCGACCCCAAGAACATCA-3′ with 5′ fluorescein and 3′ TAMRA. Target RNA levels were normalized to total RNA (Ribogreen© assessment).

### ED_50_ determination

ED_50_ values were determined with GraphPad Prism 5 software. The log dose of ASOs were plotted against mRNA level relative to untreated controls. The curves obtained were fitted using a four-parameter fit with variable slope and constraining bottom = 0 and top = 1.

### Early distribution mouse protocol

Twelve-week-old male C57BL/6 mice were administered 7.5 μmol/ kg ASO subcutaneously and sacrificed (*n* = 2/ ASO) at 0.5, 1, 2, 4, 8 and 24 h. Plasma was collected by cardiac puncture on K_2_-EDTA. Systemic tissues including heart were collected at necropsy and immediately frozen on dry ice. Concentration of ASOs in heart were determined using the protocol describe below. Immunohistochemistry analysis was done following the protocol reported in the literature (6).

### Extraction and quantitation of ASO in tissues and plasma by LCMS

ASO was extracted from plasma and tissues using previously described methods using phenol/chloroform followed by solid phase extraction using phenyl-functionalized support (25). Tissues were minced and 50–200-mg or 50–100 μl plasma samples were homogenized in 500 μl homogenization buffer (0.5% NP40 substitute (Sigma-Aldrich St. Louis, MO, USA) in Tris-buffered saline, pH 8) with Lysing Matrix D beads (MPBio Santa Ana, CA, USA) on a ball mill homogenizer (Retsch Haan, Germany) at 30 Hz for 45 s. Standard curves of each ASO were established using 500 μl aliquots of control tissue homogenate (50–200-mg tissue or 50–100 μl plasma/ml homogenization buffer). A 27-mer, fully PS, MOE/DNA oligonucleotide was added as an internal standard (IS) to all standard curves and study samples. Samples and curves were extracted with phenol/chloroform followed by solid-phase extraction (SPE) of the resulting aqueous extract using phenyl-functionalized silica sorbent (Biotage, Upsalla, Sweden). Eluate from SPE was dried down using a warm forced-air (nitrogen) evaporator and reconstituted in 100–200 μl 100 μM EDTA. Extracts were then analyzed and ASO concentration determined by LC–MS using a method similar to that described by Gaus *et al.* (26). Briefly, separation was accomplished using an 1100 HPLC–MS system (Agilent Technologies, Wilmington, DE, USA) consisting of a quaternary pump, UV detector, a column oven, an autosampler and a single quadrupole mass spectrometer. Samples were injected on an X-bridge OST C18 column (2.1 × 50 mm, 2.5-μm particles; Waters, Milford, MA, USA) equipped with a SecurityGuard C18 guard column (Phenomenex, Torrance, CA, USA). The columns were maintained at 55°C. Tributylammonium acetate buffer (5 mM) and acetonitrile were used as the mobile phase at a flow rate of 0.3 ml/min. Acetonitrile was increased as a gradient from 20 to 70% over 11 min. Mass measurements were made online using a single quadrupole mass spectrometer scanning 1000–2100 *m/z* in the negative ionization mode. Molecular masses were determined using the ChemStation analysis package (Agilent, Santa Clara, CA, USA). Manual evaluation was performed by comparing a table of calculated m/z values corresponding to potential metabolites with the peaks present in a given spectrum. Peak areas from extracted ion chromatograms were determined for ASOs and IS and a trendline established using the calibration standards, plotting concentration of ASO against the ratio of the peak areas ASO/IS. Concentration of ASOs in study samples were determined using established trendlines and reported as μg ASO/g tissue.

## RESULTS

### Palmitoyl conjugation improves plasma protein binding of cEt BNA ASO

**Figure 1.**
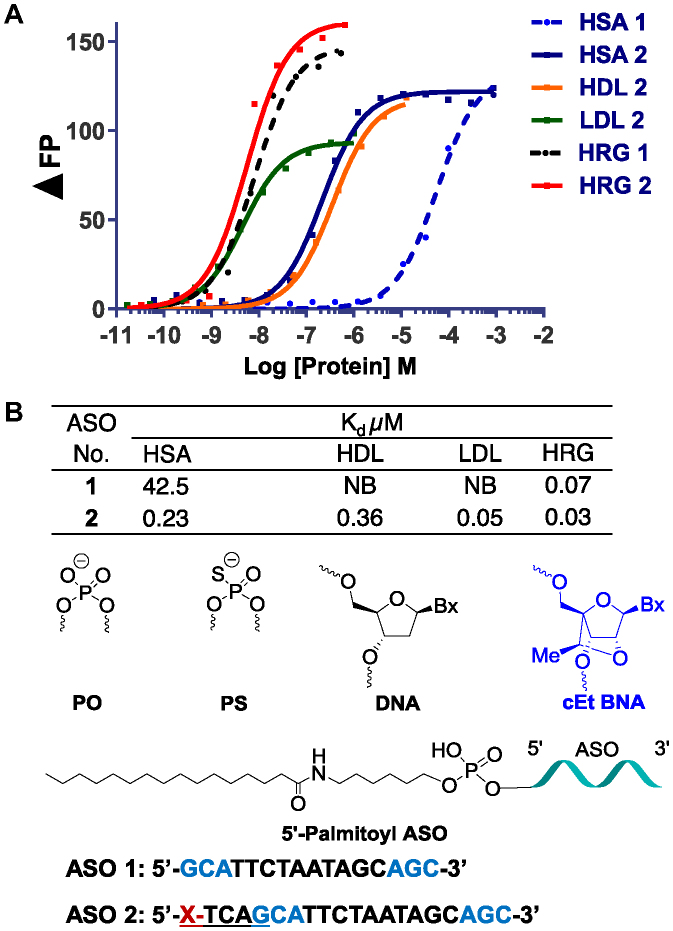
Characterizing the effect of palmitoyl conjugation on binding to selected plasma protein of Gapmer ASOs. (**A**) Binding curves showing differences between binding of unconjugated Malat-1 ASO **1** and palmitic acid conjugated ASO **2**. (**B**) Binding constants for ASO **1** and **2** to selected plasma proteins; NB: no binding; HSA: human serum albumin; HDL: high-density lipoprotein; LDL: low-density lipoprotein; HRG: histidine rich glycoprotein; ASO sequence blue: cEt BNA, black: DNA, C: 5-methylcytidine, backbone all PS; underline PO.

Phosphorothioate (PS) ASOs are known to bind plasma, cell surface and intra-cellular proteins which facilitate distribution, cellular uptake and intracellular trafficking to peripheral tissues (27). Plasma protein bound ASOs circulate transiently in the blood compartment and partition onto cell surface proteins and enter cells by endocytosis (28). Palmitic acid is known to bind to albumin and conjugation of palmitic acid to PS ASO is expected to improve albumin binding. To characterize the effect of palmitic acid conjugation on ASO binding to plasma proteins we synthesized 5′-plamitic acid conjugated 3–10–3 cEt BNA Gapmer ASO **1** (Figure 1) targeting the metastasis associated lung adenocarcinoma transcript 1 (Malat*-*1) (29). Malat*-*1 is an evolutionary conserved noncoding RNA gene highly expressed in many tissues (6). Synthesis of 5′-palmitic acid conjugated ASO **2** (Figure 1) is described in Scheme 1. The palmitoyl ASO **2** was fully characterized by ion-pair LC–MS analysis (Supporting Information).

To characterize the interaction of palmitoyl ASO **2** with albumin, we determined the binding constants using a fluorescence polarization assay (30). In addition, we also analyzed binding affinity of palmitic acid conjugates to HDL (high-density lipoproteins), LDL (low-density lipoproteins) and HRG (histidine rich glycoprotein). Palmitoyl conjugation improved the binding affinity of PS ASOs to albumin >150-fold (Figure 1). Interestingly, palmitic acid conjugation also enhanced affinity of the ASO to HDL and LDL while no change in affinity was observed for HRG (Figure 1).

### Palmitoyl conjugation improves potency of cEt BNA Malat-1 ASO 3–6-fold in muscle

**Figure 2.**
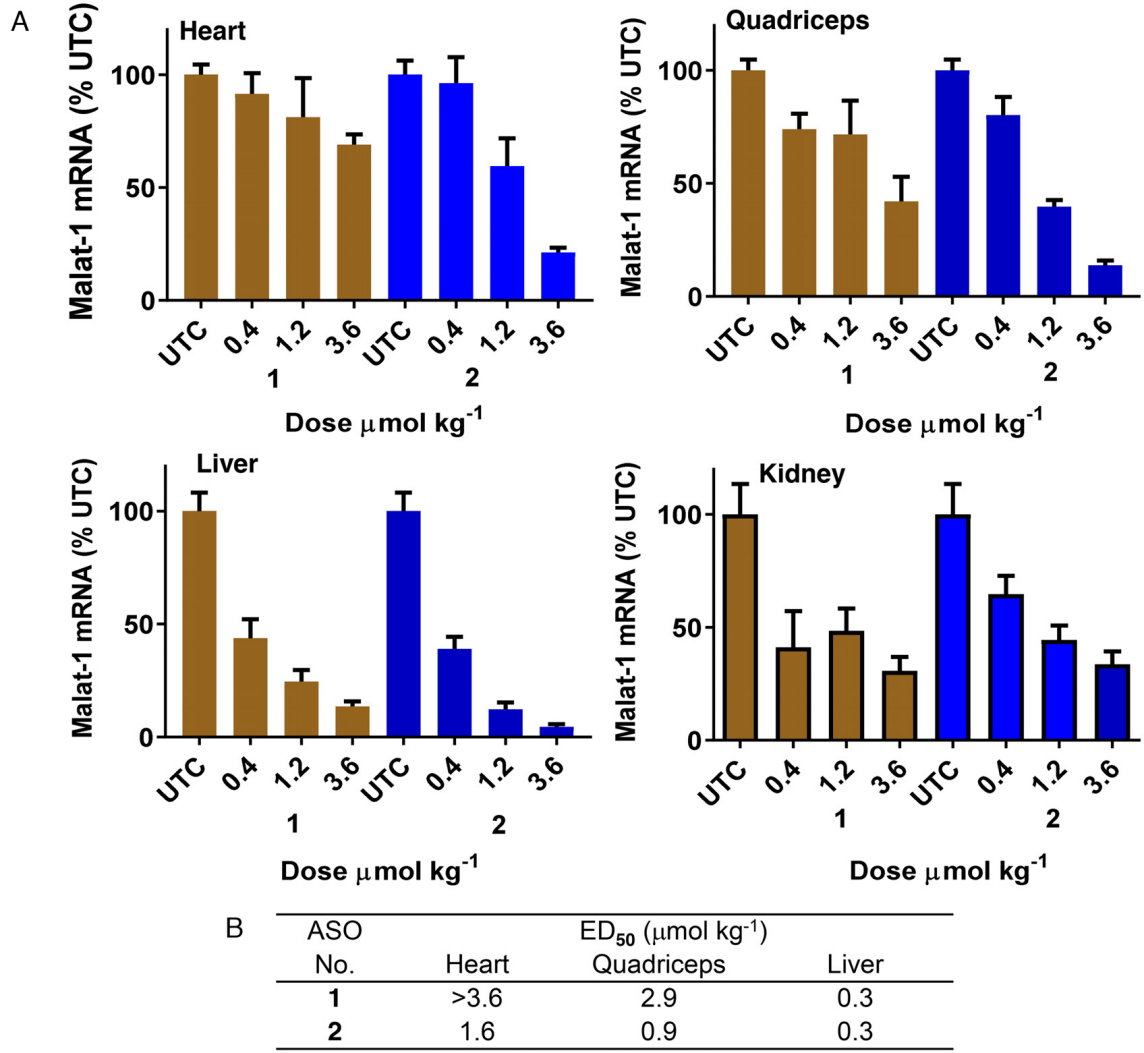
Palmitic acid conjugation enhances potency of Malat-1 ASO in mouse heart and quadriceps. Mice (C57BL/6, *n* = 4/group) were injected subcutaneously Malat-1 ASO **1** and 5′-palmitoyl conjugated Malat-1 ASO **2** at 0.4, 1.2 and 3.6 μmol/kg once a week for 3 weeks for a total of three doses and sacrificed after 5 days. (**A**) Malat-1 RNA expression analyzed in mice heart, quadriceps, liver and kidney using qRT-PCR. All data are expressed as mean ± standard deviation. (**B**) ED_50_ (μmol/kg/week) for reducing Malat-1 RNA in mouse heart, quadriceps and liver.

We next examined the effect of palmitoyl conjugation on potency of a 3–10–3 cEt BNA ASO **2** (Figure 2) targeting Malat*-*1 RNA to understand the broader effect of palmitoyl conjugation. The potency of ASO **1** and **2** to inhibit Malat-1 RNA was evaluated in mice. Mice (C57BL/6, *n* = 4/group) were injected subcutaneously with 0.4, 1.2, and 3.6 μmol/kg of ASOs **1** or **2** for 3 weeks. Five days following the last injection, mice were sacrificed and heart, quadriceps, livers and kidney were homogenized and analyzed for Malat*-*1 RNA expression. The 5′-palmitoyl Malat-1 ASO **2** showed improved potency relative to unconjugated ASO **1** in quadriceps (3-fold, Figure 2) and heart (>6 fold, Figure 2). However, no potency improvement was observed in liver and kidney. Malat-1 ASOs **1** and **2** were well tolerated with no elevations in plasma transaminases or organ weights.

The tissue concentration of Malat-1 ASOs (1,2) in heart, quadriceps and liver of treated mice were quantitated (Table 1) (31,32). Palmitoyl conjugation improved accumulation of Malat-1 ASO in heart (2–4-fold, Table 1) and quadriceps (2-fold, Table 1) relative to unconjugated ASO **1**. This data suggest that improved potency observed for palmitoyl ASO **2** is due to higher uptake of ASO in these tissues. To determine the metabolic fate of the 5′-pamitoyl ASO in heart, quadriceps and liver tissues from the mice treated with ASO **2** were homogenized and the ASO and metabolites were extracted and identified by LCMS. Interestingly, very little intact ASO **2** was extracted from tissues. The major metabolite isolated from the tissues corresponds to unconjugated ASO (data not shown) suggesting that the 5′-palmitoyl was metabolized liberating free ASO in the heart, quadriceps and liver.

**Table 1.** Concentration of Malat-1 ASO **1** and 5′-palmitoyl Malat-1 ASO **2** in the heart, quadriceps and liver

ASO No	Heart tissue concentration (μg/g)			Quadriceps tissue concentration (μg/g)			Liver tissue concentration (μg/g)		
	0.4 μmol kg^−1^	1.2 μmol kg^−1^	3.6 μmol kg^−1^	0.4 μmol kg^−1^	1.2 μmol kg^−1^	3.6 μmol kg^−1^	0.4 μmol kg^−1^	1.2 μmol kg^−1^	3.6 μmol kg^−1^
**1**	0.12 ± 0.1	0.53 ± 0.1	2.7 ± 1.2	-	0.53 ± 0.53	1.67 ± 0.33	2.76 ± 0.07	8.28 ± 1.03	23.10 ± 2.07
**2**	0.40 ± 0.1	2.13 ± 0.9	6.53 ± 2.9	-	0.80 ± 0.4	2.80 ± 1.13	4.82 ± 1.72	19.31 ± 3.45	43.45 ± 8.62

**Figure 3.**
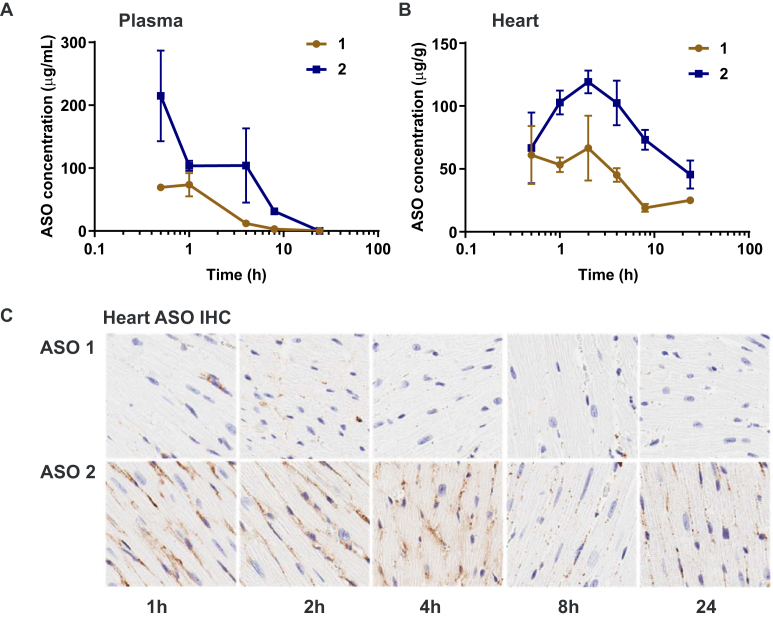
Pharmacokinetics of Malat-1 ASO **1** and 5′-palmitoyl conjugated Malat-1 ASO **2**; mice plasma total ASO concentration (**A**) and mice heart tissue total ASO concentration (**B**) after 0.5, 1, 2, 4, 8 and 24 h of subcutaneous administration of ASOs **1**–**2** (7.5 μmol kg^−1^); (**C**) ASO distribution in mice heart tissue after 1, 2, 4, 8 and 24 h of subcutaneous administration ASOs **1**–**2** (7.5 μmol kg^−1^) analyzed for ASOs by immunohistochemistry (IHC) using a PS-oligonucleotide antibody. Shown is a representative example of one of the four animals per group.

We next studied the early distribution pharmacokinetics of ASO **1** and palmitic acid conjugate ASO **2** in mice. Mice (C57BL/6) were administered 7.5 µmol/kg ASOs **1** or **2** subcutaneously and sacrificed at 0.5, 1, 2, 4, 8, and 24 h. Plasma and heart tissues were was collected and ASO concentration was analyzed by LCMS (25,26). Conjugation with palmitic acid increased plasma C_max_ of ASO nearly 3-fold, from 74 µg/ml for unconjugated ASO to 215 µg/ml for palmitate-conjugated ASO and increased plasma AUC 4-fold from 222 ± 30 µg•h/mL to 915 ± 153 µg•h/ml (Figure 3A). Similarly, C_max_ in heart increased nearly two-fold from 67- to 119 µg/g and AUC more than doubled from 682 ± 41 µg•h/g to 1676 ± 118 µg•h/g (Figure 3B) for unconjugated and palmitate-conjugated ASO respectively. Histological examination indicates that much of the ASO accumulated in heart (Figure 3C) at early time points appears diffuse or concentrated along intercellular boundaries suggesting it primarily resides in the interstitial space, however, by 24 h, the ASO appears in punctate foci, likely occupying intracellular vesicles. This increase in ASO exposure in the heart correlates with a 6-fold increase in potency of palmitoyl ASO **2** compared to unconjugated ASO **1** in the heart.

### Linker SAR to identify optimal linker strategy for palmitoyl conjugate

**Figure 4.**
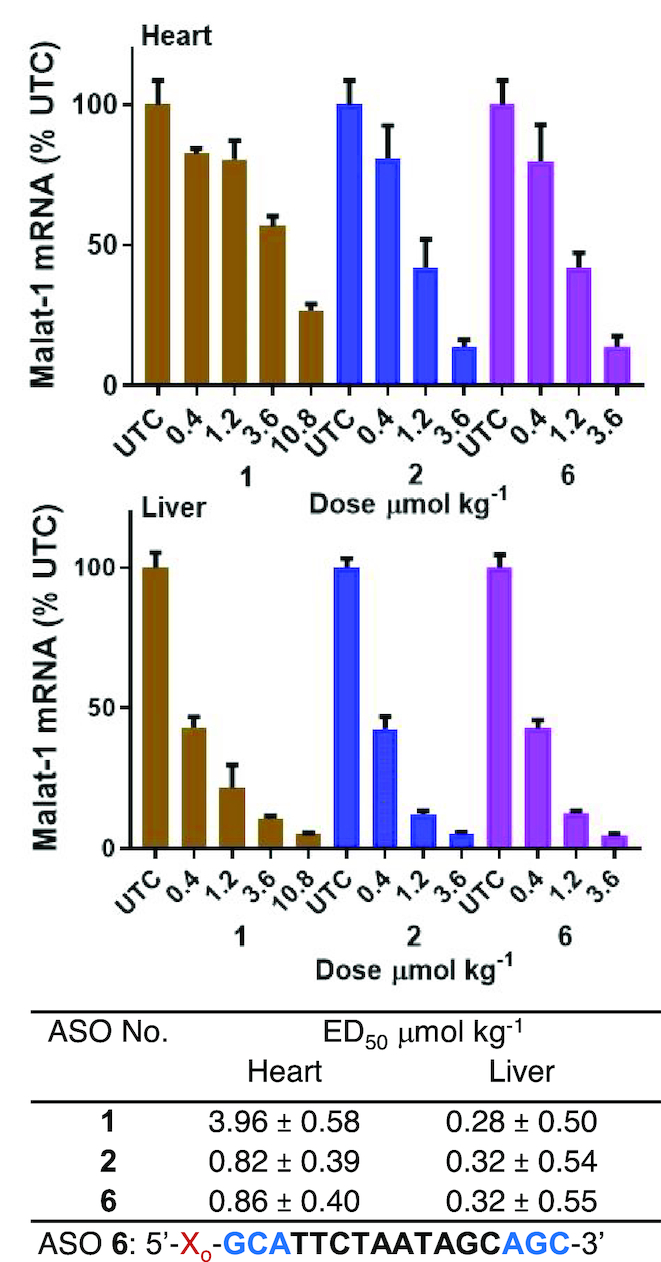
Potency of palmitic acid conjugated Malat-**1** ASO with and without PO d(TCA) linker is similar in mouse heart and liver. Mice (C57BL/6, *n* = 4/group) were injected subcutaneously ASO **1** at 0.4, 1.2, 3.6, 10.8 and ASO **2** with PO d(TCA), **6** without PO d(TCA) at 0.4, 1.2 and 3.6 μmol/kg for 3 weeks for a total of 3 doses then sacrificed after 5 days. Malat-1 RNA expression analyzed in mice heart and liver by qRT-PCR. All data are expressed as mean ± standard deviation. ED_50_ (μmol/kg/wk) for reducing Malat-1 RNA in mouse heart and ASO sequence: blue = cEt BNA, black = DNA, C = 5-methylcytidine, backbone all PS; o = PO, X = palmitoyl.

In our initial linker strategy, we attached the palmitoyl moiety using a phosphodiester d(TCA) linker which is metabolized in tissues to release the ASO. To further probe the importance of the PO d(TCA) linker moiety on potency, we evaluated ASO conjugate **6** where the fatty acid was directly attached to the ASO using a phosphodiester-linked hexylamino linker (Figure 4). 5′-Hexylamino Malat-1 ASO **7** (Scheme 1) with a PO linker was synthesized according to the reported procedure (33). The 5′-hexylamino ASO **7** was reacted with pentafluorophenyl palmitate **4** to yield ASO **6** and was fully characterized by ion-pair LC–MS analysis (Supporting Information).

Mice (C57BL/6, *n* = 4/group) were injected subcutaneously with 0.4, 1.2 and 3.6 μmol/kg of ASOs **1, 2** and **6** for three weeks. Mice were sacrificed after 5 days and heart and liver Malat*-*1 RNA expression were analyzed. ASO **2** with a PO d(TCA) (Figure 4) linker and ASO **6** with a PO linker (Figure 4) showed similar ASO activity in heart and liver. This data suggests that the PO d(TCA) linker between the fatty acid and the ASO is not required and just a PO linkage is sufficient. According to this data we selected the PO linked design chemistry for further characterization of fatty acid conjugated ASOs.

### Fatty acid structure activity relation (SAR) to identify optimal fatty acid strategy for enhancing potency of ASO in muscle and heart

**Figure 5.**
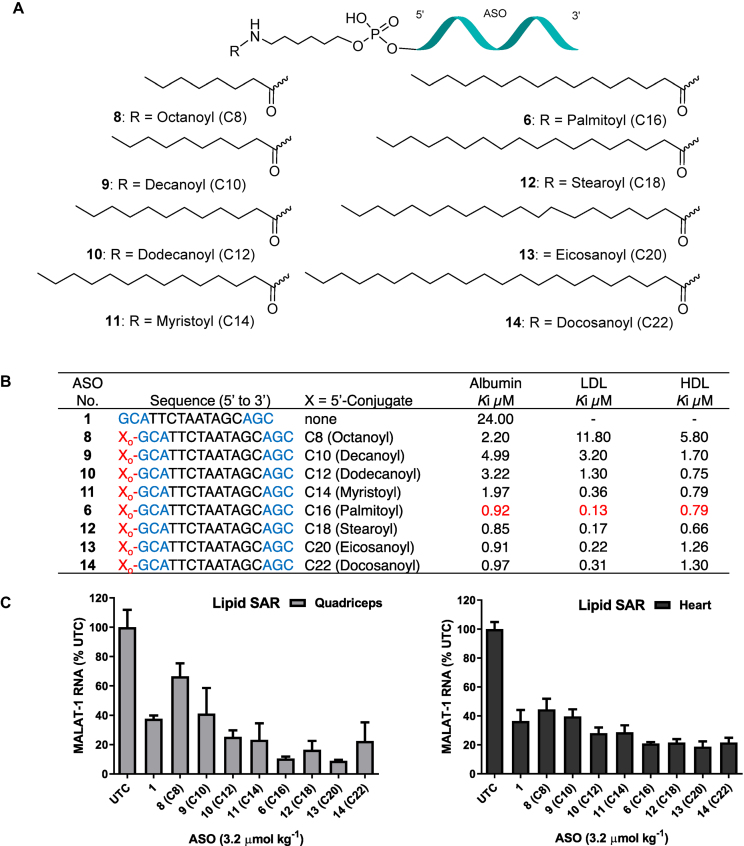
(**A**) Structures of ASO fatty acid conjugates **6, 8**–**14**; (**B**) protein binding affinity of fatty acid ASO conjugates **6, 8**–**14**; (**C**) Malat-1 RNA expression in mice heart and quadriceps after subcutaneous administration of ASO **6, 8–14** at 3.6 μmol/kg once a week for three weeks for a total of three doses; ASO sequence: X = lipid, blue: cEt BNA, black: DNA, C: 5-methylcytidine, Backbone all PS, o: PO.

Binding of fatty acids with varying chain length to albumin showed that primary association constant increased with chain length (34). Moreover, the number of high-affinity sites also increased with chain length, octanoate (C8) and decanoate (C10) bind to one site while longer chain fatty acid such as laurate (C12) and myristate (C14) bind to two sites (34). To investigate the effect of fatty acid hydrophobicity on the functional uptake of ASO we studied *in vivo* activity and plasma protein binding of eight fatty acid conjugates (Figure 5) with varying chain length.

We synthesized fatty acid conjugated ASOs **8**–**14** targeting Malat*-*1 RNA employing the similar solution phase conjugation method used for palmitoyl ASO **6** (Scheme 1). All the ASOs contain similar PO linker design as in ASO **6** without any DNA linker. Fatty acids octanoic acid **15**, decanoic acid **16**, dodecanoic acid **17**, myristic acid **18**, stearic acid **19**, eicosanoic acid **20** and docosanoic acid **21** (Scheme 1) were treated with pentafluorophenyl trifluroacetate in appropriate solvents in the presence of triethylamine at room temperature afforded corresponding pentafluorophenyl esters **22–28** (Scheme 1, Supporting Information). A solution of pentafluorophenyl esters **22–28** in appropriate solvent were added to a solution of 5′-hexylamino Malat-1 ASO **7** (Scheme 1) in sodium tetraborate buffer (pH 8.5) and the resulting solution was stirred at room temperature for 5–18 h followed by HPLC purification to provide the fatty acid conjugates ASO **8–14** (Figure 5) with varying fatty acid chain length C8-C22. Fatty acid ASO conjugates **8–14** were fully characterized by ion-pair LC–MS analysis (Supporting Information).

We examined the plasma protein binding of ASOs **8–14** using a reported fluorescence polarization (FP) competition binding assay (35) which measures the change in FP upon displacement of a 5′-palmitoyl ASO fluorophore tracer from human albumin, LDL or HDL. Interestingly, all fatty acids, regardless of chain length, improved plasma protein binding of ASO to albumin, LDL and HDL (Figure 5) relative to unconjugated ASO **1**. Albumin binding affinities of ASO fatty acid conjugates (**8-11**) with chain length C8–C14 was 2–5-fold less than ASO conjugated with chain length C16-C22 (**6, 12-14**). A similar trend was observed for the binding affinities of fatty acid conjugates **6** and **8–14** to LDL and HDL. ASO conjugates with fatty acids containing longer chain lengths of C12 to C22 (**6, 10-14**) exhibited improved binding to HDL and LDL proteins relative to ASO **8**–**9** containing fatty acid chain length C8 to C10.

We next tested the activity of ASOs **8–14** (Figure 5) for inhibiting Malat-1 RNA expression in mouse quadriceps and heart. For comparison we also evaluated the activity of unconjugated Malat-1 ASO **1** and 5′-palmitoyl conjugated ASO **6**. Mice (C57BL/6, *n* = 4/group) were injected subcutaneously with 3.6 μmol/kg of ASOs **1, 6, 8–14** for three weeks. Mice were sacrificed 5 days post-injection and Malat-1 RNA expression was analyzed from quadriceps and heart (Figure 5). ASOs conjugated to fatty acids with chain length C8 (**8**, Figure 5) and C10 (**9**, Figure 5) and unconjugated ASO **1** showed similar potency in quadriceps and heart. Interestingly, in the quadriceps and heart ASOs **10**–**14** conjugated to fatty acids with chain length from C12 to C22 were more active than ASO **1** (Figure 5). ASO conjugated to fatty acids with chain length C16 to C20 (**6, 12**–**13**, Figure 5) showed the greatest quadriceps and heart activity improvements. The ASO conjugates **6, 12**–**14** with longer fatty acid chain conjugates C16–C22 showed the highest plasma protein binding affinities. In general, albumin binding affinity appeared correlated with the activity of fatty acid conjugates in skeletal and cardiac muscle. The ASO conjugates **6, 12**–**14** with longer fatty acid chain conjugates C16–C22 showed the highest plasma protein binding affinity and *in vivo* muscle activity.

### SAR of unsaturated fatty acid ASO Conjugates

**Figure 6.**
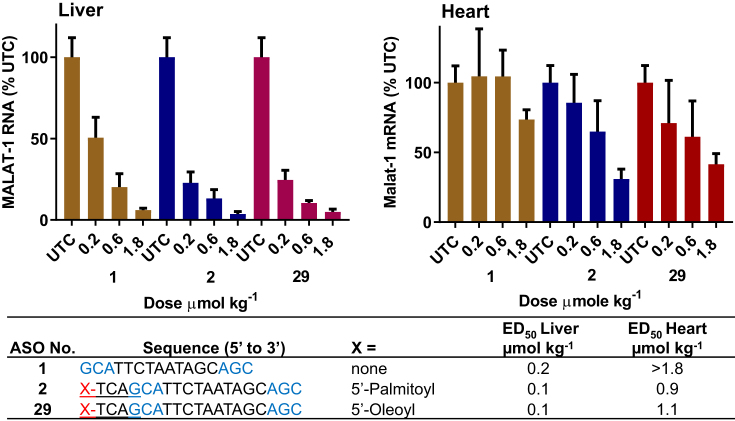
5′-Oleoyl ASO **29** and 5′-palmitoyl Malat-1 ASO **2** exhibited similar potency in mice heart and liver. Mice (C57BL/6, *n* = 4/group) were injected subcutaneously with Malat-1 ASO **1**, 5′-plamitoyl Malat-1 ASO **2** and 5′-oleyol Malat-1 ASO **29** at 0.2, 0.6 and 1.8 μmol/kg once a week for 3 weeks, sacrificed after 5 days. (**A**) Malat-1 RNA expression analyzed in mice heart and liver by qRT-PCR. All data are expressed as mean ± standard deviation. (**B**) ED_50_ (μmol/kg/week) for reducing Malat-1 RNA in mouse heart and liver. ASO sequence: X = lipid, blue: cEt BNA, black: DNA, C: 5-methylcytidine, Backbone all PS, Underline PO. (**C**) structure of 5′-oleic acid conjugated Malat-1 ASO **29**.

We also investigated the effect of conjugation of unsaturated fatty acid on muscle ASO potency. We hypothesized that unsaturated fatty acids will assume a different structure than saturated fatty acid leading to significant differences in the protein binding characteristics of unsaturated fatty acid and saturated fatty acid ASO conjugates. To test this hypothesis, we synthesized ASO **29** (Figure 6, Scheme 2) which contained an oleic acid conjugation.

**Figure 7.**
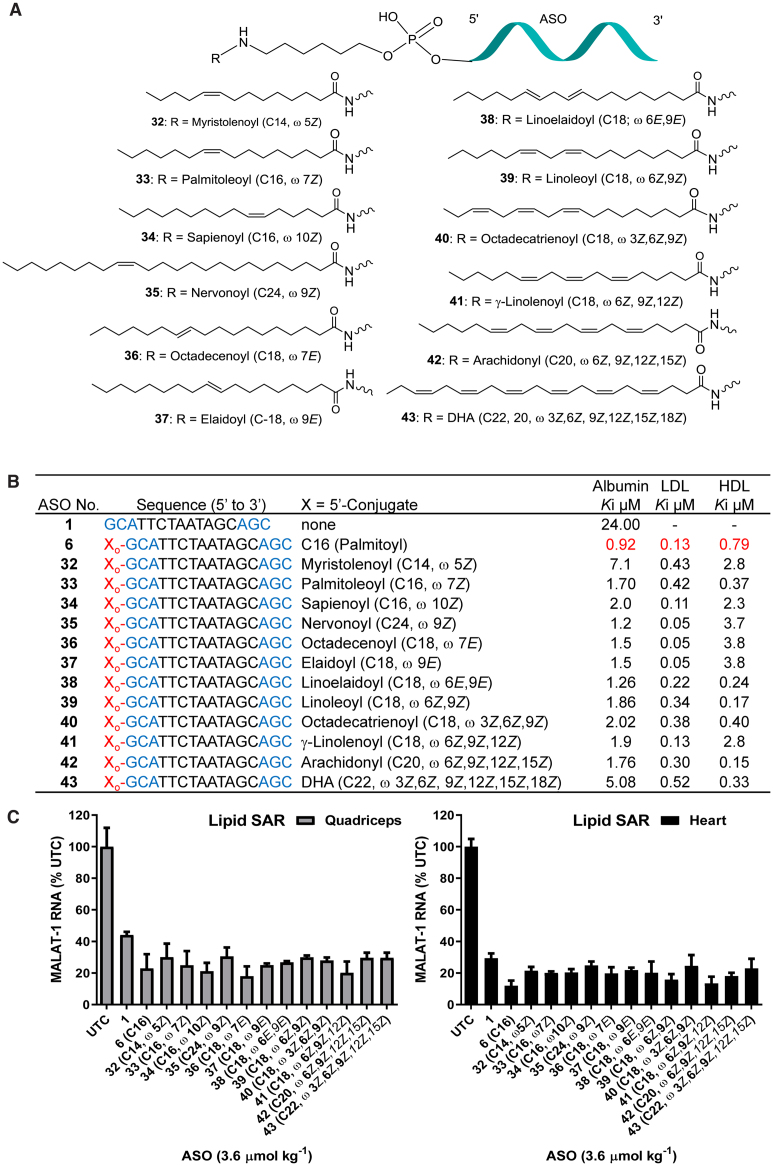
(**A**) Structure of ASO conjugates **32**–**43**; (**B**) protein binding affinity of lipid conjugates ASOs **32**–**43**; (**C**) Malat-1 RNA expression from hearts and quadriceps of ASOs **32–43** administered mice.; ASO sequence: X = lipid, blue: cEt BNA, black: DNA, C: 5-methylcytidine, Backbone all PS, o: PO.

ASO **29** (Figure 6) activity in mouse liver and heart was examined and compared with 5′-palmitoyl ASO **2** and unconjugated ASO **1**. Mice (C57BL/6, *n* = 4/group) were injected with 0.2, 0.6 and 1.8 μmol/kg of ASOs **1, 2** and **29** for three weeks. Mice were sacrificed after 5 days and Malat-1 RNA expression was analyzed from liver and heart (Figure 6). 5′-Palmitoyl ASO **2** and 5′-oleoyl ASO **29** showed similar potency in liver and heart (Figure 6). To further investigate the effect of unsaturated bond number, position, and conformation we synthesized a series of naturally occurring unsaturated fatty acid ASO conjugates and studied their activity in skeletal and cardiac muscle (Figure 7). In addition, we also characterized the plasma protein binding profile (Figure 7) of these conjugates.

We first investigated the *cis* and *trans* and positional isomers of fatty acid with one double bond. Myristolenoyl ASO **32** (Figure 7A, C14, ω 5*Z*), Palmitoleyoyl ASO **33** (Figure 7A, C16, ω 7*Z*) and sapienoyl ASO **34** (Figure 7A, C16, ω 10*Z*) with cis double bond at ω 5, 7 and 10 were synthesized (Scheme 2). To understand the effects on activity of fatty acid chain length and the degree of unsaturation we synthesized nervonoyl ASO **35** (Figure 7A, C16, ω 9*Z*) with chain length C24 and a cis double bond at ω 9. To examine the effect of double bond conformation on ASO activity we synthesized three fatty acid ASO conjugates **36**–**37** (Figure 7A) with a trans double bond at ω 7 and 9 respectively as well as linoelaidoyl ASO **38** (Figure 7A, C18, ω 6E, 9E) containing two trans double bond at ω 6 and 9. Furthermore, we also characterized the effect of conjugation of fatty acids containing 2, 3, 4 and 6 *cis* double bonds containing fatty acids on the activity and plasma protein binding of ASO. Linoleoyl ASO **39** (Figure 7A, C18, ω 6*Z*,9*Z*), octadecatrienoyl ASO **40** (Figure 7A, C18, ω 3*Z*, 6*Z*,9*Z*), γ-linolenoyl ASO **41** (Figure 7A, C18, ω 6*Z*,9*Z*,12*Z*), arachidonyl ASO **42** (Figure 7A, C20, ω 6*Z*,9*Z*,12*Z*, 15*Z*) and DHA ASO **43** (Figure 7A, C22, ω 3*Z*,6*Z*,9*Z*,12*Z*, 15*Z*, 18*Z*) were synthesized. ASOs **32**–**43** were synthesized using methods (Scheme 2) similar to the synthesis methods of other fatty acid conjugates described in this report.

Binding of unsaturated fatty acid ASO conjugates **32**–**43** to human albumin, LDL and HDL were measured using a competition binding assay (Figure 7B) (35). Comparable to saturated fatty acid ASO conjugates, all unsaturated fatty acid ASO conjugates bound to albumin, LDL and HDL (Figure 7B) with higher affinity than unconjugated ASO **1**. Interestingly, palmitoyl ASO conjugate **6** showed tighter binding to albumin, LDL and HDL relative to all unsaturated fatty acids ASO conjugates **32**–**43** assessed in this study. Consistent with previous observation, binding affinity of unsaturated fatty acid conjugates with shorter chain length (ASO **32**, C14 ω 5*Z*) was less than longer chain unsaturated fatty acids. Double bond confirmation and position appears to have minimum effect of the plasma protein binding of ASO conjugates. ASOs **33**–**35** containing cis double bond at different position and ASOs conjugates **36**–**38** with trans double bonds at different positions showed similar plasma protein binding affinity (Figure 7B). Fatty acid conjugates containing different degrees of unsaturation exhibited interesting albumin binding affinity trends. ASO conjugate **43** with six cis unsaturated double bonds bound to albumin (*K*_i_ 5 μM) at a lower affinity compared to ASOs **32–35** and **39**–**42** (*K*_i_ 1.7–2 μM) with one to four *cis* double bonds.

To assess the effect of structural variation of fatty acid on activity, we evaluated the activity of ASO conjugates **32**–**43** for inhibiting Malat-1 RNA expression in mice and compared it to 5′-palmitoyl ASO **6** and unconjugated ASO **1**. Mice (C57BL/6, *n* = 4/group) were subcutaneously injected with 3.6 μmol/kg of ASOs **1, 6** and **32**–**43** for three weeks. Mice were sacrificed after 5 days and Malat-1 RNA expression was analyzed from quadriceps and heart (Figure 7C). All fatty acid conjugates ASOs showed improved activity in quadriceps and heart compared to unconjugated ASO **1** (Figure 7C). However, 5′-palmitoyl ASO **6** exhibited improved activity compared to all other unsaturated fatty acid conjugated ASOs examined in the study (Figure 7C). It appears that geometry or number of unsaturation do not significantly influence the functional uptake of unsaturated fatty acid conjugates. ASOs **32**–**35** containing fatty acids with cis double bond at different position and ASOs **36**–**38** containing trans double bonds showed similar activity. Similarly, ASO conjugates **39**–**43** containing two to six *cis* double bonds also showed similar activity in muscle. Consistent with saturated fatty acid conjugates, plasma protein binding affinity correlated with the skeletal and cardiac muscle activity of unsaturated fatty acid ASO conjugates

### Palmitoyl conjugation improves potency of cEt BNA CD36 ASO 3–7 fold in heart and muscle

**Figure 8.**
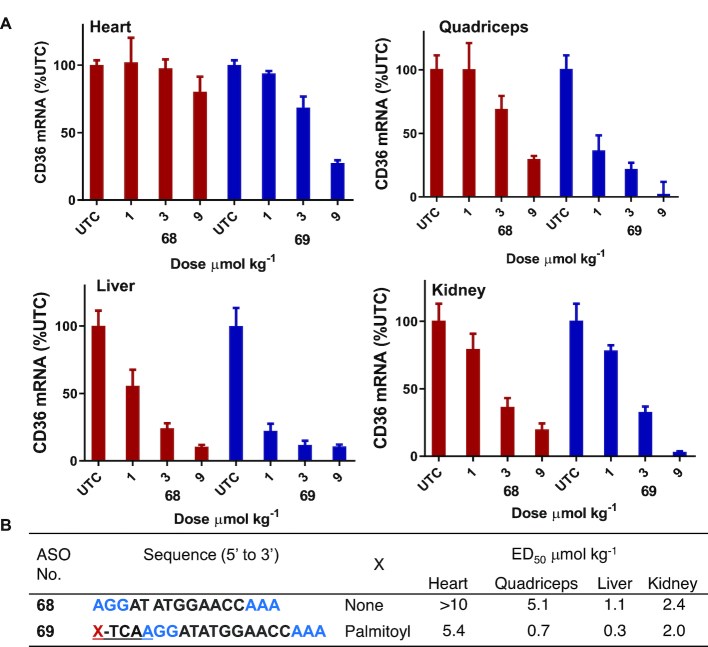
Palmitic acid conjugation enhances potency of CD36 ASO in mice heart, quadriceps and, liver. Mice (C57BL/6, *n* = 4/group) were injected intravenously CD36 ASO **68**, 5′-plamitoyl CD36 ASO **68** at 1, 3 and 9 μmol/kg once a week for 3 weeks, sacrificed after 5 days. (**A**) CD36 mRNA expression analyzed in mice hearts quadriceps, liver and kidney by qRT-PCR. All data are expressed as mean ± standard deviation. (**B**) ED_50_ (μmol/kg/week) for reducing CD36 mRNA in mouse heart, quadriceps, liver and kidney. ASO sequence: X = lipid, blue: cEt BNA, black: DNA, C: 5-methylcytidine, backbone all PS, underline PO.

Next, we examined the effects of palmitic acid conjugation on the potency of ASO targeting CD36 mRNA in mouse heart and quadriceps. CD36 ASO **68** (Figure 8) is a fully PS modified cEt BNA Gapmer ASO targeting CD36 mRNA, which is expressed in both murine liver and extra-hepatic tissues (36). Activity of ASOs targeting mouse CD36 mRNA have been previously characterized (36). To study the effect of palmitic acid modification on activity of CD36 ASO we synthesized 5′-palmitoyl conjugated CD36 ASO **69** (Figure 8) using the method described in Scheme 1.

Mice (C57BL/6, *n* = 4/group) were injected intravenously with 1, 3 and 9 μmol/kg of ASOs **68** or **69** for 3 weeks. Mice were sacrificed 5 days post-injection and, heart, quadriceps, liver and kidney were homogenized and analyzed for reduction of CD36 mRNA. Palmitoyl ASO **69** showed more than 7-fold enhanced potency in quadriceps and heart relative to the parent ASO **68** (Figure 8). In liver, potency improvement was only 3-fold whereas in kidney similar potency was exhibited with unconjugated ASO and palmitic acid conjugated ASO. All ASOs were well tolerated with no elevations in plasma transaminases or organ weights (data not shown).

### Palmitoyl conjugation enhances potency of ASO targeting DMPK mRNA in mice

**Figure 9.**
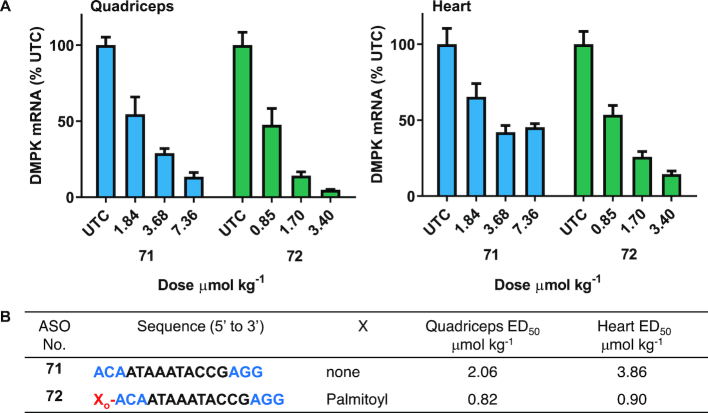
Palmitic acid conjugation enhances potency of DMPK ASO in mice heart and quadriceps. Mice (Bal/c, *n* = 4/group) were administered subcutaneously DMPK ASO **71** at 1.84, 3.68 and 7.36 and 5′-palmitoyl DMPK ASO **72** at 0.85, 1.70, 3.40, μmol/kg once a week for 3.5 weeks, sacrificed after 2 days. (**A**) DMPK mRNA expression analyzed in mice heart and quadriceps by qRT-PCR. All data are expressed as mean ± standard deviation. (**B**) ED_50_ (μmol/kg/week) for reducing DMPK mRNA in mouse heart and quadriceps. ASO sequence: X = lipid, blue: cEt BNA, black: DNA, C: 5-methylcytidine, backbone all PS, o = PO.

Myotonic dystrophy type 1 (DM1) is a neuromuscular diseases caused by inherited CTG repeat expansion in the gene encoding DM Protein Kinase (DMPK) (7). The CTG repeats in the gene are transcribed into mRNA which cause hairpins to form and bind with high affinity to the muscle blind-like (MBNL) family of proteins. This complex is sequestered and prevents them from performing their normal function (7). In preclinical studies, antisense oligonucleotides targeted to DMPK mRNA efficiently reduced mRNA in different skeletal muscle (37,38) which led to improvements in body weight, muscle strength and muscle histology (38). However, in the clinic the DMPK ASO exhibited limited therapeutic benefit (7), indicating an urgent need for improved potency. In this report we evaluated the potency of a previously characterized (37) DMPK ASO **71** (Figure 9) in muscle and show that palmitoyl conjugation improves the potency of DMPK ASO in mice quadriceps and heart (Figure 9).

Mice (*n* = 4/group) were injected subcutaneously with DMPK ASOs **71**at 1.84, 3.68 and 7.36 μmol/kg and **72** at 0.85, 1.70 and 3.40 μmol/kg per week for 4 weeks. Mice were sacrificed 2 days following their last dose and quadriceps and heart DMPK mRNA expression was analyzed (Figure 9). Palmitoyl modification led to a 3–4-fold improvement of DMPK ASO **72** activity in quadriceps and heart relative to unconjugated ASO **71** (Figure 9).

### Palmitoyl conjugation enhances potency of ASO targeting Cav3 mRNA more than 5-fold in mice skeletal and cardiac muscle

**Figure 10.**
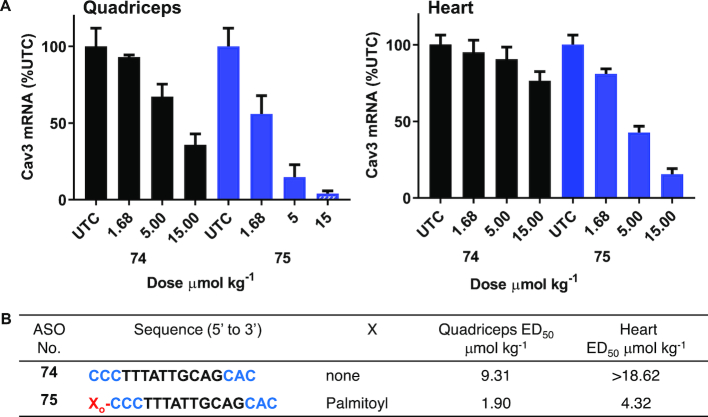
Activity of Cav3 ASO **74** and 5′-palmitoyl Cav3 ASO **75** in mice heart and quadriceps; Mice (C57BL/6, *n* = 4/group) were injected subcutaneously Cav3 ASO **74**, 5′-palmitoyl Cav3 ASO **75** at 1.68, 5 and 15 μmol/kg once a week for 2 weeks, sacrificed after 4 days. (**A**) Cav3 mRNA expression analyzed in mice heart and quadriceps by qRT-PCR. All data are expressed as mean ± standard deviation. (**B**) ED_50_ (μmol/kg/week) for reducing Cav3 mRNA in mouse heart and quadriceps. ASO sequence: X = lipid, blue: cEt BNA, black: DNA, C: 5-methylcytidine, backbone all PS, o = PO.

Caveolin 3 (Cav3) is a caveolin family member protein expressed in cell types that act as a scaffolding protein for the organization and concentration of certain caveolin-interacting molecules. Mutations in this gene is attributed to lead to Limb-Girdle muscular dystrophy type-1C (LGMD-1C), hyperCKemia, or rippling muscle disease (RMD) (39). It has been reported that Cav3 expression in mice is primarily in heart and skeletal muscle (40). We designed antisense oligonucleotide **74** (Figure 10) targeting Cav3 mRNA and demonstrated modest activity in quadriceps and heart. To evaluate the effect of palmitic acid conjugation on potency of Cav3 ASO we synthesized 5′-palmitoyl conjugated Cav 3 ASO **75** (Figure 10, Scheme 1) and studied activity in mice.

We then evaluated the potency of ASOs **74**–**75** to inhibit Cav3 mRNA in mice quadriceps and heart. Mice (*n* = 4/group) were injected subcutaneously with ASOs **74** or **75** at 1.68, 5 and 15 μmol/kg per week for two weeks. Mice were sacrificed 4 days following the final dose and Cav3 mRNA expression was analyzed from quadriceps and heart (Figure 10). Palmitoyl Cav3 ASO **75** exhibited improved potency in quadriceps (5-fold) and in heart (> 4-fold) compared to unconjugated ASO **74** (Figure 10). This data further confirms that that palmitoyl conjugation is a worthwhile approach to improve the potency of clinically relevant ASO in mice.

### Solid phase synthesis of 5′-palmitic acid conjugated ASOs

**Scheme 3.**
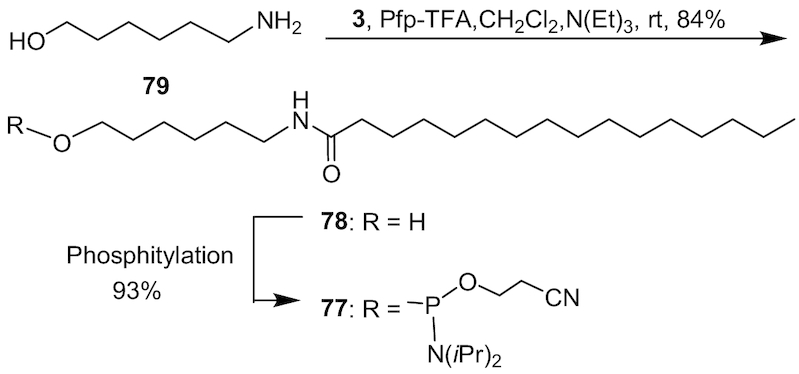
Synthesis of 6-palmitamidohexyl phosphoramidite **77**; Pfp-TFA: pentaflurophenyl trifluoroacetate.

We developed a solid phase DNA synthesis method for convenient synthesis of 5′-palmitic acid conjugated ASOs. First, we developed a 6-palmitamidohexyl phosphoramidite **77** (Scheme 3) for the incorporation of palmitic acid modification using a solid phase DNA synthesis method. Phosphoramidite **77** was synthesized according to the method described in Scheme 3. In brief, 6-palmitamidohexanol **78** (84%) was generated from palmitic acid, Pfp-TFA, triethylamine and 6-aminohexanol **79** in dichloromethane in a one pot synthesis. Compound **78** was subsequently phosphitylated to afford phsophoramidite **77** (93%). 5′-Pamitic acid conjugated ASO were synthesized on a solid phase DNA synthesizer using phosphoramidite **77** using standard protocol in good yield (29).

## DISCUSSION

Antisense oligonucleotide therapeutics is a maturing drug discovery platform with six ASO drugs approved for clinical use (3,41,42), and over 40 more in clinical development (3). Given the predominant distribution of ASOs to the liver, it is not surprising that the majority of systemically administrated ASOs in clinical use or development target the liver-expressed genes. Thus, delivery strategies will be essential to define a new class of RNA therapeutics that target extrahepatic sites such as muscle. We demonstrate that fatty acid conjugation can improve potency of RNase H ASOs 3 to7 fold for suppressing gene expression in skeletal and cardiac muscles.

**Figure 11.**
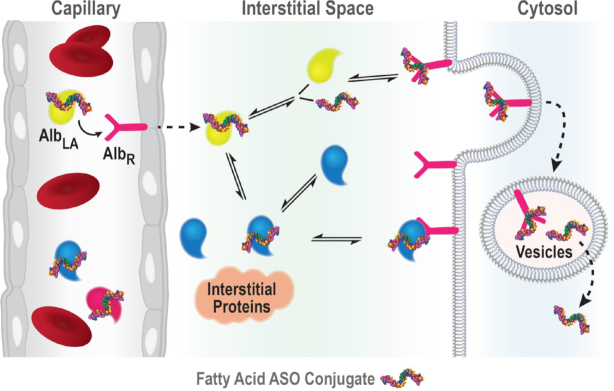
Schematic representation of the likely route taken by lipid conjugated ASO from the capillary to cytosol in muscle. AlbLA: albumin bound lipid ASO; AlbR: albumin receptor.

Fatty acids constitute a fundamental source of energy production (43). The majority of fatty acids in the plasma are bound to serum albumin and there are seven binding sites for long-chain non-esterified fatty acids on the albumin (44). Albumin is one of the most abundant proteins in plasma and provides the transport of fatty acids, drugs, ions and other metabolites (45). Albumin interacts with multiple receptors such as glycoprotein Gp60, Gp30 and Gp18, (SPARC), the Megalin/Cublin complex, and the neonatal Fc receptor (FcRn) (20). Albumin's interaction with these receptors is responsible for its recycling and cellular transcytosis (20). This property makes albumin an attractive ‘self’ drug delivery molecules to transit across tissues and cellular barriers (20). Transport from circulation to muscle cells ASO must pass the endothelium, the interstitial space, and the plasma membrane (Figure 11). We hypothesized that conjugation of fatty acid will increase the albumin binding affinity of ASOs enhancing their ability to cross the endothelia barrier and improve its functional uptake into muscles (Figure 11). Previous studies suggested that unconjugated PS ASOs bind to human serum albumin with low binding affinity 370–480 μM (46). A recent report suggests that fatty acid modified gapmer ASOs can self-assemble into constructs that offer improved tissue distribution (47).

As expected, palmitoyl conjugation improved binding affinity of ASO to human serum albumin (Figure 1) >150-fold. While, palmitic acid modification enhanced binding affinity of ASO to other most prominent plasma proteins such as HDL, and LDL but binding affinity to HRG remained unchanged (Figure 1). We next studied the effect of palmitic acid conjugation on potency of Malat-1 ASOs in mice quadriceps, heart, liver and kidney. Interestingly, palmitic acid conjugation improved the potency of Malat-1 ASO more than five-fold in heart and quadriceps (Figure 2) after systemic administration. In kidney unconjugated and palmitoyl conjugated Malat-1ASOs exhibited similar potency. In addition, palmitoyl conjugation improved tissue uptake of Malat-1 ASO in heart and quadriceps (Table 1) thus providing a rationale for the enhanced potency. Interestingly, palmitic acid conjugation increased plasma Cmax of ASO and plasma AUC relative to unconjugated ASO. Histological examination indicates that much of the ASO accumulated in heart at early time points appears diffuse or concentrated along intercellular boundaries suggesting it primarily resides in the interstitial space, however, by 24 h, the ASO appears in punctate foci, likely occupying intracellular vesicles. These data provide initial evidence for improved delivery of ASO when conjugated to palmitic acid in the muscle interstitial space compared to unconjugated ASO.

One of the principal functions of human serum albumin is to transport fatty acids. The primary association constant of albumin to fatty acids is a function of chain length and degree of unsaturation (34), where the primary association constant and number of high affinity sites increase with fatty acid chain length (34). We characterized the effect of fatty acid chain length and degree of unsaturation on the albumin binding affinity of fatty acid conjugated ASOs. Binding affinity increased with chain length and the optimum affinity was observed in ASO conjugates with C16-C18 fatty acid chain length. Double bond unsaturation number or stereochemistry had little effect on albumin binding affinity of the ASO conjugate. Fatty acid conjugates with one to four unsaturated double bonds in cis conformation and with one or two trans double bond exhibited similar albumin binding. Interestingly, ASO conjugates containing C16 to C18 saturated or unsaturated fatty acids exhibited similar activity in mice skeletal and cardiac muscle. Taken together, our data suggest that palmitoyl (C16 saturated) and oleoyl (C18, ω 9*Z*) conjugation provided maximum potency enhancement for ASOs in skeletal and cardiac muscle.

We also demonstrate that potency improvement observed for palmitoyl conjugation in mouse skeletal and cardiac muscle is not sequence or target specific. Palmitic acid conjugation improved potency (3 to 7 fold) of ASOs targeting CD36, DMPK and Cav3 mRNA in mouse muscle compared to corresponding unconjugated ASOs. The unconjugated DMPK ASO was evaluated in clinical trials for the treatment of myotonic dystrophy type 1.

Our data demonstrate that altering the interaction of PS ASO with specific plasma proteins could modulate the tissue distribution of PS ASO. Conjugation of palmitic acid, a known albumin ligand, enhanced the albumin affinity of PS ASO. Albumin is actively transported across the capillary endothelium *via* caveolin-1 mediated transcytosis and significant amounts (60%) of total albumin is associated with the interstitial space in muscle (48). We hypothesize fatty acid conjugation enhances albumin binding thereby facilitating ASO movement across the vascular wall into the interstitium. Additional work is needed to understand more completely this process, as well as whether myocyte delivery itself is also enhanced by fatty acid interactions on plasma membrane receptors.

In conclusion, we report a detailed structure-activity relationship of fatty acid conjugated ASOs for harnessing albumin-based delivery into extrahepatic tissues. Activity and binding to plasma proteins of ASO fatty acid conjugates containing varying fatty acid chain length, degree of unsaturation and cis trans isomers were studied. The binding affinity of ASO to plasma proteins improved with fatty acid chain length with the highest binding affinity observed with fatty acid chain length from 16 to 18 carbons. Degree of unsaturation or conformation of double bond appears to have no influence on protein binding of ASO fatty acid conjugates. Activity of fatty acid ASO conjugates correlate with the affinity to albumin and the tightest albumin binder exhibited highest activity improvement in muscle. Palmitic and oleic acid appears to be the optimum fatty acid structure for improving functional uptake of ASO into skeletal and cardiac muscle. To access the site of action in muscle, ASO must traverse the vascular wall and enter the interstitium to have access to the myocyte. Our data suggest that fatty acid conjugation enables the ASO to bind tightly to albumin thereby facilitating ASO delivery to the interstitium leading to enhanced myocyte uptake and ASO activity. Further improvement in effectiveness of fatty acid ASO conjugates could be achieved by combining with ligands which target cell-surface receptors in muscle tissues. Strategy described in this report provides a foundation for designing more effective therapeutic ASOs for targeting muscle tissues to develop treatments for muscle related diseases.

## Supplementary Material

gkz354_Supplemental_Files
