# Supplementary material for: Fatty acid conjugation enhances potency of antisense oligonucleotides in muscle
Source: Nucleic Acids Res. 2019 May 25;47(12):6029–44. doi: 10.1093/nar/gkz354 (PMC6614804; doi:10.1093/nar/gkz354)
Supplement: gkz354_Supplemental_Files [file gkz354_supplemental_files.docx]

**Supplementary Data**

**Fatty acid conjugation enhances potency of antisense oligonucleotides in muscle**

Thazha P. Prakash*, Adam E. Mullick, Richard Lee, Jinghua Yu, Steve T. Yeh, Audrey Low, Alfred E. Chappell, Michael E. Oestergaard, Sue Murray, Hans J. Gaus, Eric E. Swayze, Punit P. Seth

*Ionis Pharmaceuticals, 2855 Gazelle Ct., Carlsbad, CA 92010, USA*

*Corresponding author Email:tprakash@ionisph.com*

**Table of Contents**

| I | Table S1 sequence and analytical data for ASOs | S2 |
| --- | --- | --- |
| II | Experimental for synthesis of fatty acid-Pfp esters **4**, **22**-**28**, **31**, **56**-**67** | S3-S6 |
| III | Experimental for synthesis of 5’—hexylamino ASOs 5, 7, 70, 73, 76 | S6-S7 |

**Table S1.** Sequence and analytical data for fatty acid conjugated ASOs

| ASO No. | Sequence (5’-3’) | X = | Calcd Mass | Found Mass | %UV Purity |
| --- | --- | --- | --- | --- | --- |
| **1** | **GCATTCTAATAGCAGC** | - | 5414.6 | 5413.6 | 90.23 |
| **2** | X-TCA**GCATTCTAATAGCAGC** | 5’-Palmitoyl | 6752.8 | 6751.4 | 91.86 |
| **6** | X-**GCATTCTAATAGCAGC** | 5’-Palmitoyl | 5832.2 | 5832.0 | 94.99 |
| **8** | X-**GCATTCTAATAGCAGC** | 5’-Octanoyl | 5720.0 | 5719.7 | 93.10 |
| **9** | X-**GCATTCTAATAGCAGC** | 5’-Decanoyl | 5748.0 | 5747.7 | 95.49 |
| **10** | X-**GCATTCTAATAGCAGC** | 5’-Dodecanoyl | 5776.1 | 5775.9 | 93.85 |
| **11** | X-**GCATTCTAATAGCAGC** | 5’-Myristoyl | 5804.1 | 5804.4 | 97.54 |
| **12** | X-**GCATTCTAATAGCAGC** | 5’-Stearoyl | 5860.2 | 5859.6 | 98.32 |
| **13** | X-**GCATTCTAATAGCAGC** | 5’-Eicosanoyl | 5887.8 | 5887.8 | 95.18 |
| **14** | X-**GCATTCTAATAGCAGC** | 5’-Docosanoyl | 5916.3 | 5916.4 | 97.80 |
| **29** | X-TCA**GCATTCTAATAGCAGC** | 5’-Oleioyl | 6778.8 | 6777.3 | 92.10 |
| **32** | X-**GCATTCTAATAGCAGC** | 5’-Myristolenoyl | 5802.1 | 5801.7 | 97.37 |
| **33** | X-**GCATTCTAATAGCAGC** | 5’-Palmitoleoyl | 5830.1 | 5829.9 | 96.19 |
| **34** | X-**GCATTCTAATAGCAGC** | 5’-Sapienoyl | 5830.2 | 5829.9 | 97.65 |
| **35** | X-**GCATTCTAATAGCAGC** | 5’-Nervonoyl | 5942.4 | 5942.0 | 96.47 |
| **36** | X-**GCATTCTAATAGCAGC** | 5’-Octadecenoyl | 5858.2 | 5858.8 | 98.15 |
| **37** | X-**GCATTCTAATAGCAGC** | 5’-Elaidoyl | 5858.2 | 5857.2 | 97.05 |
| **38** | X-**GCATTCTAATAGCAGC** | 5’-Linoelaidoyl | 5856.2 | 5856.0 | 87.14 |
| **39** | X-**GCATTCTAATAGCAGC** | 5’-Linoleoyl | 5856.2 | 5855.7 | 95.64 |
| **40** | X-**GCATTCTAATAGCAGC** | 5’-Octadecatrienoyl | 5853.8 | 5853.6 | 84.40 |
| **41** | X-**GCATTCTAATAGCAGC** | 5’-γ-Linolenoyl | 5854.2 | 5854.4 | 92.72 |
| **42** | X-**GCATTCTAATAGCAGC** | 5’-Arachidonyl | 5880.2 | 5878.5 | 82.65 |
| **43** | X-**GCATTCTAATAGCAGC** | 5’-DHA | 8563.1 | 8562.8 | 80.42 |
| **68** | **AGGAT ATGGAACCAAA** | - | 5468.6 | 5467.5 | 93.33 |
| **69** | **X-TCAAGGATATGGAACCAAA** | 5’-Palmitoyl | 6806.8 | 6806.0 | 93.39 |
| **71** | **ACAATAAATACCGAGG** | - | 5442.6 | 5441.7 | 97.16 |
| **72** | **X-TCAACAATAAATACCGAGG** | 5’-Palmitoyl | 5860.2 | 5859.2 | 99.22 |
| **74** | **CCCTTTATTGCAGCAC** | - | 5369.6 | 5367.9 | 90.60 |
| **75** | **X-TCACCCTTTATTGCAGCAC** | 5’-Palmitoyl | 5,787.2 | 5786.9 | 96.07 |

ASO sequence: X = lipid, blue: cEt BNA, black: DNA, C: 5-methylcytidine, backbone all PS, Underline: PO.

**Synthesis of fatty acid pentafluorophenyl esters 4, 22-28, 31, 56-67**. Synthesis was accomplished using the general method for the synthesis of pentafluorophenyl esters described in the manuscript. All compounds were well characterized by 1H, 13C and 19F NMR analysis. Fatty acid pentafluorophenyl esters were found to be unstable under mass spectrometry analysis could not obtained the mass for these compounds.

**Compound 4.** (16.1 g, 97%, white solid). ^1^H NMR (300MHz, CDCl_3_) δ: 2.67 (t, *J* = 7.4 Hz, 2H), 1.78 (m, 2H), 1.49-1.16 (m, 24H), 0.89 (t, *J* = 7.4 Hz, 3H); ^19^F NMR (282MHz, CDCl_3_) δ: -152.81 to -152.94 (m, 2F), -158.34 (t, *J* = 21.7 Hz, 1F), -162.44 to -162.64 (m, 2F); ^13^C NMR (75MHz, CDCl_3_) δ: 169.6, 143.0, 141.1, 139.6, 137.7, 136.2, 125.4, 33.4, 31.9, 29.7, 29.7, 29.7, 29.6, 29.6, 29.4, 29.1, 28.9, 24.8, 22.7, 14.1.

**Compound 22.** (1.08 g, quantitative, clear oil). ^1^H NMR (300MHz, CDCl_3_) δ: 2.67 (t, *J* = 7.4 Hz, 1H), 1.79 (t, *J* = 7.4 Hz, 2H), 1.51 - 1.20 (m, 8H), 0.91 (t, *J* = 7.4 Hz, 3H); ^19^F NMR (282MHz, CDCl_3_) δ: -152.84 to -152.97 (m, 2F), -158.36 (t, *J* = 21.7 Hz, 1F), -162.46 to -162.66 (m, 2F)**;** ^13^C NMR (75MHz, CDCl_3_) δ: 169.6, 142.8, 141.1, 139.5, 137.7, 136.2, 125.2, 33.4, 31.6, 28.8, 28.8, 24.8, 22.6, 14.0.

**Compound 23.** (0.96 g, 98%). ^1^H NMR (300MHz, CDCl_3_) δ: 2.67 (t, *J* = 7.4 Hz, 2H), 1.78 (m, 2H), 1.51-1.11 (m, 12H), 0.89 (t, *J* = 7.4 Hz, 3H); ^19^F NMR (282MHz, CDCl_3_) δ: -152.81 to -152.92 (m, 2F), -158.33 (t, *J*=21.7 Hz, 1F), -162 to -162.63 (m, 2F);^13^C NMR (75MHz, CDCl_3_) δ: 169.6, 142.9, 141.1, 139.6, 137.7, 136.3, 125.2, 33.4, 31.8, 29.3, 29.2, 29.1, 28.9, 24.8, 22.6, 14.1.

**Compound 24.** (1.04 g, 96%, clear oil). ^1^H NMR (300MHz, CDCl_3_) δ: 2.67 (t, *J* = 7.4 Hz, 2H), 1.78 (m, 2H), 1.49-1.22 (m, 16H), 0.89 (t, *J* = 7.4 Hz, 3H); ^19^F NMR (282MHz, CDCl_3_) δ: -152.81 to -152.93 (m, 2F), -158.33 (t, *J* = 21.7 Hz, 1F), -162.43 to -162.63 (m, 2F); ^13^C NMR (75MHz, CDCl_3_) δ: 169.6, 142.9, 141.1, 139.6, 137.7, 136.3, 125.2, 33.4, 31.9, 29.6, 29.5, 29.4, 29.3, 29.1, 28.9, 24.8, 22.7, 14.1.

**Compound 25.** (1.01 g, 97%, clear oil). ^1^H NMR (300MHz, CDCl_3_) δ: 2.67 (t, *J* = 7.4 Hz, 2H), 1.78 (m, 2H), 1.50-1.15 (m, 20H), 0.89 (t, *J* = 7.4 Hz, 3H); ^19^F NMR (282MHz, CDCl_3_) δ: -152.82 to -152.93 (m, 2F), -158.34 (t, *J* = 21.7 Hz, 1F), -162.43 to -162.64 (m, 2F); ^13^C NMR (75MHz, CDCl_3_) δ: 169.6, 142.8, 141.1, 139.6, 137.7, 136.2, 125.2, 33.4, 31.9, 29.7, 29.6, 29.6, 29.6, 29.4, 29.1, 28.9, 24.8, 22.7, 14.1.

**Compound 26**. (0.99 g, 96%, white sold). ^1^H NMR (300MHz, CDCl_3_) δ: 2.67 (t, *J* = 7.4 Hz, 2H), 1.78 (m, 2H), 1.49-1.16 (m, 28H), 0.89 (t, *J* = 7.4 Hz, 3H); ^19^F NMR (282MHz, CDCl_3_) δ: -152.82 to -152.94 (m, 2F), -158.33 (br t, *J* = 21.7 Hz, 1F), -162.44 to -162.62 (m, 2F); ^13^C NMR (75MHz, CDCl_3_) δ: 169.6, 142.9, 141.1, 139.6, 137.7, 136.3, 125.2, 33.3, 31.9, 29.7, 29.6, 29.5, 29.4, 29.1, 28.9, 24.8, 22.7, 14.07.

**Compound 27.** (1.52 g, 99%, white sold). ^1^H NMR (300MHz, CDCl_3_) δ: 2.67 (t, *J* = 7.4 Hz, 2H), 1.78 (m, 2H), 1.50-1.14 (m, 32H), 0.89 (t, *J* = 7.4 Hz, 2H);^19^F NMR (282MHz, CDCl_3_) δ: -152 to -152.94 (m, 2F), -158.35 (t, *J* = 21.7 Hz, 1F), -162.44 to -162.65 (m, 2F)**;** ^13^C NMR (75MHz, CDCl_3_) δ: 169.6, 142.9, 141.1, 139.6, 139.4, 136.3, 125.2, 33.4, 31.9, 29.7, 29.6, 29.4, 29.4, 29.1, 28.9, 24.8, 22.7, 14.1.

**Compound 28.** (1.48 g, quantitative, white sold). ^1^H NMR (300MHz, CDCl_3_) δ: 2.67 (t, *J* = 7.4 Hz, 2H), 1.78 (m, 2H), 1.52 - 1.15 (m, 36H), 0.89 (brt, *J* = 7.4 Hz, 3H); ^19^F NMR (282MHz, CDCl_3_) δ: -152.80 to -152.90 (m, 2F), -158.31 (br t, *J* = 21.7 Hz, 1F), -162.43 to -162.61 (m, 2F); ^13^C NMR (75MHz, CDCl_3_) δ: 169.6, 142.9, 141.1, 139.6, 139.4, 136.3, 125.2, 33.4, 31.9, 29.7, 29.6, 29.5, 29.4, 29.3, 29.1, 28.9, 24.8, 22.7, 14.1.

**Compound 31** (3.07 g, 98.1%, clear oil). ^1^H NMR (300MHz, CDCl_3_) δ: 5.44-5.31 (m, 2H), 2.67 (t, *J* = 7.4 Hz, 2H), 2.13-1.94 (m, 4H), 1.78 (m, 2H), 1.50-1.17 (m, 20H), 0.96-0.79 (m, 3H); ^19^F NMR (282MHz, CDCl_3_) δ: -152.82 to -152.94 (m, 2F), -158.25 (t, *J*=21.7 Hz, 1F), -162.42 to -162.63 (m, 2F); ^13^C NMR (75MHz, CDCl_3_) δ: 169.53, 142.80, 141.08, 139.57, 137.90, 136.19, 130.09, 129.66, 125.23, 33.34, 31.90, 29.76, 29.63, 29.51, 29.33,29.31, 29.03, 29.01, 28.84, 27.23, 27.13, 24.76, 22.67, 14.07.

**Compound 56.** (0.33 g, 96%, clear oil). ^1^H NMR (300MHz, CDCl_3_) δ: 5.50-5.17 (m, 2H), 2.67 (t, *J* = 7.4 Hz, 2H), 2.18-1.93 (m, 4H), 1.78 (m, 2H), 1.51-1.25 (m, 12H), 0.91 (t, *J* = 7.4 Hz, 3H); ^19^F NMR (282MHz, CDCl_3_) δ: -152.82 to -152.94 (m, 2F), -158.32 (t, *J* = 21.7 Hz, 1F), -162.42 to -162.62 (m, 2F); ^13^C NMR (75MHz, CDCl_3_) δ: 169.6, 142.9, 141.1, 139.6, 137.7, 136.2, 130.0, 129.7, 125.2, 33.3, 32.0, 29.6, 29.0, 29.0, 28.8, 27.1, 26.9, 24.8, 22.3, 14.0.

**Compound 57.** (1.65 g, quantitative, clear oil). ^1^H NMR (300MHz, CDCl_3_) δ: 5.55-5.13 (m, 2H), 2.67 (t, *J* = 7.4 Hz, 2H), 2.15-1.94 (m, 4H), 1.78 (m, 2H), 1.51-1.19 (m, 16H), 0.89 (t, *J* = 7.4 Hz, 3H); ^19^F NMR (282MHz, CDCl_3_) δ: -152.81 to -152.94 (m, 2F), -158.32 (t, *J* = 21.7 Hz, 1F), -162.42 to -162.62 (m, 2F); ^13^C NMR (75MHz, CDCl_3_) δ: 169.5, 142.8, 141.1, 139.5, 137.7, 136.2, 130.1, 129.7, 125.2, 33.3, 31.8, 29.7, 29.6, 29.0, 28.8, 27.2, 27.1, 24.8, 22.7, 14.1.

**Compound 58.** (0.14 g, 86%, clear oil). ^1^H NMR (300MHz, CDCl_3_) δ: 5.47-5.31 (m, 2H), 2.68 (t, *J* = 7.4 Hz, 2H), 2.14–2.00 (m, 4H), 1.85-1.75 (m, 2H), 1.56-1.44 (m, 2H), 1.41-1.16 (m, 14H), 0.89 (t, *J* = 7.4 Hz, 3H); ^19^F NMR (282MHz, CDCl_3_) δ: -152.82 to -152.93 (m, 2F), -158.28 (t, *J* = 21.7 Hz, 1F), -162.41 to -162.59 (m, 2F); ^13^C NMR (75MHz, CDCl_3_) δ: 169.4, 142.9, 141.1, 139.6, 137.7, 136.3, 130.9, 128.6, 125.2, 33.3, 31.9, 29.7, 29.6, 29.3, 28.9, 27.3, 26.7, 24.4, 22.7, 14.1.

**Compound 59.** (0.27 g, 92%, clear oil). ^1^H NMR (300MHz, CDCl_3_) δ: 5.41-5.31 (m, 2H), 2.67 (t, *J*=7.4 Hz, 2H), 2.02 (q, *J* = 6.2 Hz, 4H), 1.78 (ap. quin, *J* = 7.4 Hz, 2H), 1.50 - 1.15 (m, 32H), 0.89 (t, *J* = 7.4 Hz, 3H); ^19^F NMR (282MHz, CDCl_3_) δ: -152.82 to -152.93 (m, 2F), -158.32 (t, *J* = 21.7 Hz, 1F), -162.41 to -162.62 (m, 2F); ^13^C NMR (75MHz, CDCl_3_) δ: 169.6, 142.8, 141.1, 139.6, 139.4, 136.2, 129.9, 125.0, 33.4, 31.9, 29.8, 29.7, 29.6, 29.5, 29.4, 29.3, 29.1, 28.9, 27.2, 24.8, 22.7, 14.1.

**Compound 60.** (0.3 g, 94%, clear oil). ^1^H NMR (300MHz, CDCl_3_) δ: 5.46-5.33 (m, 2H), 2.67 (t, *J* = 7.4 Hz, 2H), 2.06-1.92 (m, 4H), 1.78 (m, 2H), 1.50-1.23 (m, 20H), 0.89 (t, *J* = 7.4 Hz, 3H); ^19^F NMR (282MHz, CDCl_3_) δ: -152.81 to -152.94 (m, 2F), -158.33 (t, *J*=21.7 Hz, 1F), -162.43 to -162.63 (m, 2F); ^13^C NMR (75MHz, CDCl_3_) δ: 169.6, 142.8, 141.1, 139.6, 137.7, 136.2, 130.4, 130.3, 125.2, 33.4, 32.6, 31.8, 29.6, 29.4, 29.3, 29.1, 28.9, 28.8, 24.8, 22.6, 14.1.

**Compound 61**. (1.52 g, 96%, clear oil). ^1^H NMR (300MHz, CDCl_3_) δ: 5.48-5.33 (m, 2H), 2.67 (t, *J* = 7.4 Hz, 2H), 1.99 (brs, 4H), 1.78 (m, 2H), 1.49-1.24 (m, 20H), 0.89 (t, *J* = 7.4 Hz, 3H); ^19^F NMR (282MHz, CDCl_3_) δ: -152 to -152.93 (m, 2F), -158.32 (t, *J*=21.7 Hz, 1F), -162.42 to -162.62 (m, 2F); ^13^C NMR (75MHz, CDCl_3_) δ: 169.6, 142.8, 141.0, 139.5, 137.7, 136.2, 130.6, 130.1, 125.2, 33.3, 32.6, 32.5, 31.9, 29.7, 29.5, 29.3, 29.2, 29.0, 28.9, 28.8, 24.8, 22.7, 14.1.

**Compound 62.** (0.38 g, 96%, clear oil). ^1^H NMR (300MHz, CDCl_3_) δ: 5.49-5.35 (m, 4H), 2.75 - 2.61 (m, 4H), 2.10-1.94 (m, 4H), 1.78 (m, 2H), 1.50-1.20 (m, 14H), 0.89 (t, *J* = 7.4 Hz, 3H); ^19^F NMR (282MHz, CDCl_3_) δ: -152.82 to -152.93 (m, 2F), -158.31 (br t, *J* = 21.7 Hz, 1F), -162.41 to -162.61 (m, 2F); ^13^C NMR (75MHz, CDCl_3_) δ: 169.6, 142.8, 141.0, 139.6, 137.7, 136.2, 131.1, 130.8, 128.8, 128.5, 125.2, 35.6, 33.3, 32.5, 32.5, 31.4, 29.4, 29.2, 29.0, 28.9, 28.8, 24.8, 14.0.

**Compound 63.** (1.55 g, 97%, clear oil). ^1^H NMR (300MHz, CDCl_3_) δ: 5.47-5.29 (m, 4H), 2.79 (t, *J* = 5.7 Hz, 2H), 2.67 (t, *J* = 7.4 Hz, 2H), 2.18-1.97 (m, 4H), 1.78 (m, 2H), 1.52-1.15 (m, 14H), 0.90 (t, *J* = 5.7 Hz, 3H); ^19^F NMR (282MHz, CDCl_3_) δ: -152.81 to -152.93 (m, 2F), -158.29 (t, *J* = 21.7 Hz, 1F), -162.40 to -162.60 (m, 2F); ^13^C NMR (75MHz, CDCl_3_) δ: 169.5, 142.9, 141.1, 139.6, 137.7, 136.2, 130.2, 130.0, 128.1, 127.9, 125.2, 33.3, 31.5, 29.5, 29.3, 29.0, 28.8, 27.2, 27.1, 25.6, 24.8, 22.6, 14.0.

**Compound 64.** (0.8 g, quantitative, clear oil). ^1^H NMR (300MHz, CDCl_3_) δ: 5.51-5.20 (m, 6H), 2.82 (t, *J* = 5.8 Hz, 4H), 2.67 (t, *J* = 7.4 Hz, 2H), 2.09 (m, 4H), 1.79 (m, 2H), 1.52-1.28 (m, 8H), 0.99 (t, *J* = 7.5 Hz, 3H); ^19^F NMR (282MHz, CDCl_3_) δ: -152.83 to -152.96 (m, 2F), -158.28 (t, *J* = 21.6 Hz, 1F), -162.40 to -162.61 (m, 2F); ^13^C NMR (75MHz, CDCl_3_) δ: 169.6, 142.9, 141.1, 139.5, 139.7, 136.2, 132.0, 130.2, 128.3, 128.2, 127.8, 127.1, 33.3, 29.5, 29.0, 28.8, 27.2, 25.6, 25.5, 24.8, 20.5, 14.2.

**Compound 65.** (0.28 g, 89%, clear oil). ^1^H NMR (300MHz, CDCl_3_) δ: 5.49-5.30 (m, 6H), 2.90-2.79 (m, 4H), 2.68 (t, *J* = 7.4 Hz, 2H), 2.24-2.11 (m, 2H), 2.07 (q, *J* = 6.7 Hz, 2H), 1.90-1.73 (m, 2H), 1.61 - 1.45 (m, 2H), 1.43-1.20 (m, 6H), 0.90 (t, *J* = 7.4 Hz, 3H); ^19^F NMR (282MHz, CDCl_3_) δ: -152.80 to -152.93 (m, 2F), -158.24 (t, *J* = 21.7 Hz, 1F), -162.36 to -162.57 (m, 2F); ^13^C NMR (75MHz, CDCl_3_) δ: 169.4, 142.9, 141.1, 139.6, 137.7, 136.2, 130.5, 129.2, 128.6, 128.5, 128.0, 127.6, 125.2, 33.2, 31.5, 29.3, 28.8, 27.2, 26.7, 25.7, 24.4, 22.6, 14.0.

**Compound 66.** (1.5 g, 97%, clear oil). ^1^H NMR (300MHz, CDCl_3_) δ: 5.53-5.29 (m, 8H), 2.90-2.80 (m, 6H), 2.69 (t, *J* = 7.4 Hz, 2H), 2.23 (q, *J* = 7.3 Hz, 2H), 2.06 (q, *J* = 6.7 Hz, 2H), 1.87 (m, 2H), 1.45 - 1.21 (m, 6H), 0.90 (t, *J* = 7.4 Hz, 3H); ^19^F NMR (282MHz, CDCl_3_) δ: -152.78 to -152.91 (m, 2F), -158.19 (t, *J* = 21.7 Hz, 1F), -162.31 to -162.52 (m, 2F); ^13^C NMR (75MHz, CDCl_3_) δ: 169.4, 142.8, 141.1, 139.5, 137.8, 136.2, 130.5, 128.6, 128.4, 128.2, 127.9, 127.8, 127.5, 125.1, 32.6, 31.5, 29.3, 27.2, 26.3, 25.6, 25.6, 24.6, 22.6, 14.0.

**Compound 67.** (1.0 g, 65%, light brown oil). ^1^H NMR (300MHz, CDCl_3_) δ: 5.67-5.27 (m, 12H), 2.94 - 2.79 (m, 10H), 2.78-2.66 (m, 2H), 2.55 (q, *J* = 7.0 Hz, 2H), 2.15-2.05 (m, 2H), 0.98 (t, *J* = 7.6 Hz, 3H); ^19^F NMR (282MHz, CDCl_3_) δ: -152.62 to -152.74 (m, 2F), -158.12 (t, *J* = 21.7 Hz, 1F), -162.30 to -162.50 (m, 2F); ^13^C NMR (75MHz, CDCl_3_) δ: 168.9, 142.8, 141.1, 139.5, 137.8, 136.2, 132.0, 130.4, 128.6, 128.5, 128.3, 128.0, 127.9, 127.8, 127.0, 126.6, 125.1, 33.3, 25.6, 25.5, 22.6, 20.5, 14.2.

**Compound 60.** (0.3 g, 94%, clear oil). ^1^H NMR (300MHz, CDCl_3_) δ: 5.46-5.33 (m, 2H), 2.67 (t, *J* = 7.4 Hz, 2H), 2.06-1.92 (m, 4H), 1.78 (m, 2H), 1.50-1.23 (m, 20H), 0.89 (t, *J* = 7.4 Hz, 3H); ^19^F NMR (282MHz, CDCl_3_) δ: -152.81 to -152.94 (m, 2F), -158.33 (t, *J* = 21.7 Hz, 1F), -162.43 to -162.63 (m, 2F); ^13^C NMR (75MHz, CDCl_3_) δ: 169.6, 142.8, 141.1, 139.6, 137.7, 136.2, 130.4, 130.3, 125.2, 33.4, 32.6, 31.8, 29.6, 29.4, 29.3, 29.1, 28.9, 28.8, 24.8, 22.6, 14.1.

**Compound 61.** (1.52 g, 96%, clear oil). ^1^H NMR (300MHz, CDCl_3_) δ: 5.48-5.33 (m, 2H), 2.67 (t, *J* = 7.4 Hz, 2H), 1.99 (brs, 4H), 1.78 (m, 2H), 1.49-1.24 (m, 20H), 0.89 (t, *J* = 7.4 Hz, 3H); ^19^F NMR (282MHz, CDCl_3_) δ: -152 to -152.93 (m, 2F), -158.32 (t, *J*=21.7 Hz, 1F), -162.42 to -162.62 (m, 2F); ^13^C NMR (75MHz, CDCl_3_) δ: 169.6, 142.8, 141.0, 139.5, 137.7, 136.2, 130.6, 130.1, 125.2, 33.3, 32.6, 32.5, 31.9, 29.7, 29.5, 29.3, 29.2, 29.0, 28.9, 28.8, 24.8, 22.7, 14.1.

**Compound 62.** (0.38 g, 96%, clear oil). ^1^H NMR (300MHz, CDCl_3_) δ: 5.49-5.35 (m, 4H), 2.75 - 2.61 (m, 4H), 2.10-1.94 (m, 4H), 1.78 (m, 2H), 1.50 - 1.20 (m, 14H), 0.89 (t, *J* = 7.4 Hz, 3H); ^19^F NMR (282MHz, CDCl_3_) δ: -152.82 to -152.93 (m, 2F), -158.31 (br t, *J* = 21.7 Hz, 1F), -162.41 to -162.61 (m, 2F); ^13^C NMR (75MHz, CDCl_3_) δ: 169.6, 142.8, 141.0, 139.6, 137.7, 136.2, 131.1, 130.8, 128.8, 128.5, 125.2, 35.6, 33.3, 32.5, 32.5, 31.4, 29.4, 29.2, 29.0, 28.9, 28.8, 24.8, 14.0.

**General Procedure for synthesis of 5’-hexylamino ASOs 5, 7, 70, 73, 76.** Antisense oligonucleotides were synthesized at 40 μmol scale using UnyLinker™ solid support. cEt BNA nucleoside phosphoramidites were synthesized using reported procedures.[^1^](#_ENREF_1) 0.1 M solution of 2’-cEt BNA nucleoside phosphoramidites in 40% dichloromethane in acetonitrile was used for respective ASO synthesis. A solution of DNA nucleoside phosphoramidites in acetonitrile (0.1 M), and standard oxidizing and capping reagents were used. For each of the modified analogs 4-fold excess of modified nucleoside 3’-phosphoramidite were delivered with a 12 min coupling time. For 5’-hexylamino modification a solution (0.1 M) 5’-Amino-Modifier C6 from Glen Research (22825 Davis Drive, Sterling-20164, Virginia, U. S. A.) in acetonitrile containing 4-methoxytrityl (MMT) group protected amine. The MMT group was left on to facilitate purification. Post-synthetically, all oligonucleotides were treated with 1:1 triethylamine: acetonitrile to remove cyanoethyl protecting groups from the PS or PO linkages. Subsequently, solid support bearing ASOs were treated with aqueous NH_4_OH (28-30 wt%) at room temperature for 4 h and then added 10% (V/V) of 40% methylamine in water. Continued ageing of the solution at room temperature for additional 18-20 h to cleave 5’-(*N*-MMT)-hexylamino ASOs from support, remove protecting groups, and hydrolyze the UnyLinker™ moiety. To this triethylamine (10% V/V) was added and ammonia was boiled off from the solution by bubbling nitrogen. ASOs were purified by HPLC on a reverse-phase column (Waters, Delta Pak, 300 x 19 mm, C4, 15 μm, 300 Ä, Buffer A: 0.1 M ammonium acetate, Buffer B; acetonitrile, 0 to 60% B in 60 min, flow 4 mL min^-1^, λ 260 nm). Fractions containing full length ASOs were pooled together and pH was adjusted to 5.0 (add solid sodium acetate to get 1.5 M sodium acetate solution) and heated at 45 ^o^C for 60 min for removing MMT group. Monitored the reaction by LCMS. Expected mass obtained. Adjusted pH to 7 by 1 M sodium hydroxide. Diluted with water and desalted using HPLC on a reverse phase column. Purity and mass of oligonucleotides were determined using ion-pair LCMS analysis.

REFERENCES

1. Seth, P. P.; Vasquez, G.; Allerson, C. A.; Berdeja, A.; Gaus, H.; Kinberger, G. A.; Prakash, T. P.; Migawa, M. T.; Bhat, B.; Swayze, E. E. Synthesis and Biophysical Evaluation of 2',4'-Constrained 2'o-Methoxyethyl and 2',4'-Constrained 2'o-Ethyl Nucleic Acid Analogues. *J. Org. Chem.* **2010,** 75, 1569-1581.
